# Molecular Encapsulation of Naphthalene Diimide (NDI) Based π‐Conjugated Polymers: A Tool for Understanding Photoluminescence

**DOI:** 10.1002/anie.202110139

**Published:** 2021-10-18

**Authors:** Jeroen Royakkers, Kunping Guo, Daniel T. W. Toolan, Liang‐Wen Feng, Alessandro Minotto, Daniel G. Congrave, Magda Danowska, Weixuan Zeng, Andrew D. Bond, Mohammed Al‐Hashimi, Tobin J. Marks, Antonio Facchetti, Franco Cacialli, Hugo Bronstein

**Affiliations:** ^1^ Department of Chemistry University of Cambridge Lensfield Road Cambridge CB2 1EW UK; ^2^ Cavendish Laboratory University of Cambridge Cambridge CB3 0HE UK; ^3^ Department of Physics and Astronomy and LCN University College London Gower Street London WC1E 6BT UK; ^4^ Department of Chemistry University of Sheffield Brook Hill Sheffield S3 7HF UK; ^5^ Department of Chemistry Northwestern University 2145 Sheridan road Evanston IL 60208-3113 USA; ^6^ Department of Chemistry Texas A&M University at Qatar P.O. Box 23874 Doha Qatar

**Keywords:** conjugated polymers, encapsulation, macrocycles, organic electronics, photoluminescence

## Abstract

Conjugated polymers are an important class of chromophores for optoelectronic devices. Understanding and controlling their excited state properties, in particular, radiative and non‐radiative recombination processes are among the greatest challenges that must be overcome. We report the synthesis and characterization of a molecularly encapsulated naphthalene diimide‐based polymer, one of the most successfully used motifs, and explore its structural and optical properties. The molecular encapsulation enables a detailed understanding of the effect of interpolymer interactions. We reveal that the non‐encapsulated analogue **P(NDI‐2OD‐T)** undergoes aggregation enhanced emission; an effect that is suppressed upon encapsulation due to an increasing π‐interchain stacking distance. This suggests that decreasing π‐stacking distances may be an attractive method to enhance the radiative properties of conjugated polymers in contrast to the current paradigm where it is viewed as a source of optical quenching.

## Graphical abstract

This article demonstrates a novel synthetic strategy that can be used to tune the interstrand distance between conjugated polymers with unprecedented precision. This control provides an ideal platform for fundamental insight into the solid‐state optical properties of NDI polymers.

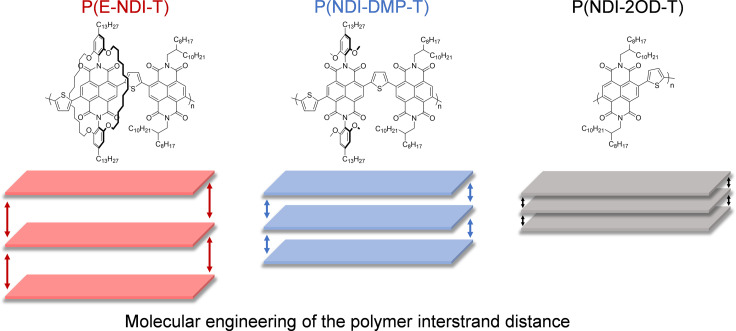

## Introduction

π‐Conjugated polymers are an important class of semiconductors with unique and tunable optoelectronic properties, which hold great promise for the development of solution‐processible and low‐cost sustainable energy applications, such as organic photovoltaics (OPVs), organic light‐emitting diodes (OLEDs), organic field‐effect transistors (OFETs), sensors and energy storage devices.[^1^, ^2^]

In particular, naphthalene diimide (NDI) based π‐conjugated polymers are among the most interesting electron‐transporting semiconductors given their remarkable track record as high‐performance non‐fullerene acceptors (NFAs) in OPVs[^2^, ^3^, ^4^, ^5^] and high mobility n‐channel semiconductors in OFETs.[^2^, ^6^] NDI's excellent device performance and many favorable properties (e.g., appropriate energy levels, facile synthesis, low synthetic cost, good electron mobility, high solubility as well as thermal and (photo)chemical stability) have therefore made this electron‐depleted core extremely popular in plastic electronics.[^2^, ^7^, ^8^, ^9^] However, since NDI‐based polymers such as N2200 [P(NDI‐2OD‐2T)] or PCE8 [P(NDI‐2OD‐T)] are based on quite large aromatic units, they have a great propensity to aggregate in both solution and as thin films.^[5]^ This can directly affect the conjugated polymer properties (both optical and electronic) as well as their miscibility with other polymers and hence greatly influences their device performance. Gaining greater synthetic control over the intermolecular interactions of these materials is therefore very important but remains challenging.

Recently, it has become apparent that one of the most important obstacles that must be overcome in conjugated polymer design is the suppression of non‐radiative decay in narrow band gap conjugated polymers.^[10]^ Numerous recent studies have revealed that the high non‐radiative voltage loss encountered in organic photovoltaics (OPVs), which holds back their efficiency, is primarily a consequence of the low solid state photoluminescence quantum yields (PLQYs) of the blend materials.[^11^, ^12^] Hence, increasing the PLQY of narrow band gap conjugated polymers is essential for next generation OPV, as well as being appealing for light emitting diodes and biological imaging. It is commonly assumed that the close packing of conjugated polymers results in the creation of additional non‐radiative decay pathways. Therefore, the most promising strategy to increase the solid‐state PLQY of conjugated polymers is to molecularly isolate the chains either through covalent or non‐covalent encapsulation.[^13^, ^14^, ^15^, ^16^, ^17^, ^18^, ^19^, ^20^, ^21^] Polymer dilution in a host matrix can also be used to increase PLQY but this cannot suppress intramolecular interactions, or even intermolecular in some cases.^[22]^ Herein, we report the synthesis and characterization of a novel encapsulated conjugated NDI polymer and report the resulting effect of molecular encapsulation on its optical properties. For our study we chose to encapsulate a P(NDI‐T) derivative as it is one of the most successful n‐type conjugated polymers used in organic photovoltaics.^[4]^ Thus, our findings are applicable and of relevance to virtually all polymer‐polymer solar cell devices. We present a novel synthetic methodology which represents a significant advance in the functionalization of NDI‐based monomers and allows for a much wider use of solubilizing/bulky functional groups on the N‐atom of the naphthalene diimide. This strategy enables the tuning of intermolecular interactions in NDI‐based polymers in an unprecedented fashion, and thereby allows invaluable fundamental insight into their solid‐state optical properties. Our key finding is that upon increasing conjugated polymer inter‐strand separation, the photoluminescence is quenched due to the suppression of an AIEE (aggregation induced enhanced emission) type phenomena which is present in the parent polymer.

## Results

### Synthesis

**Figure 1:**
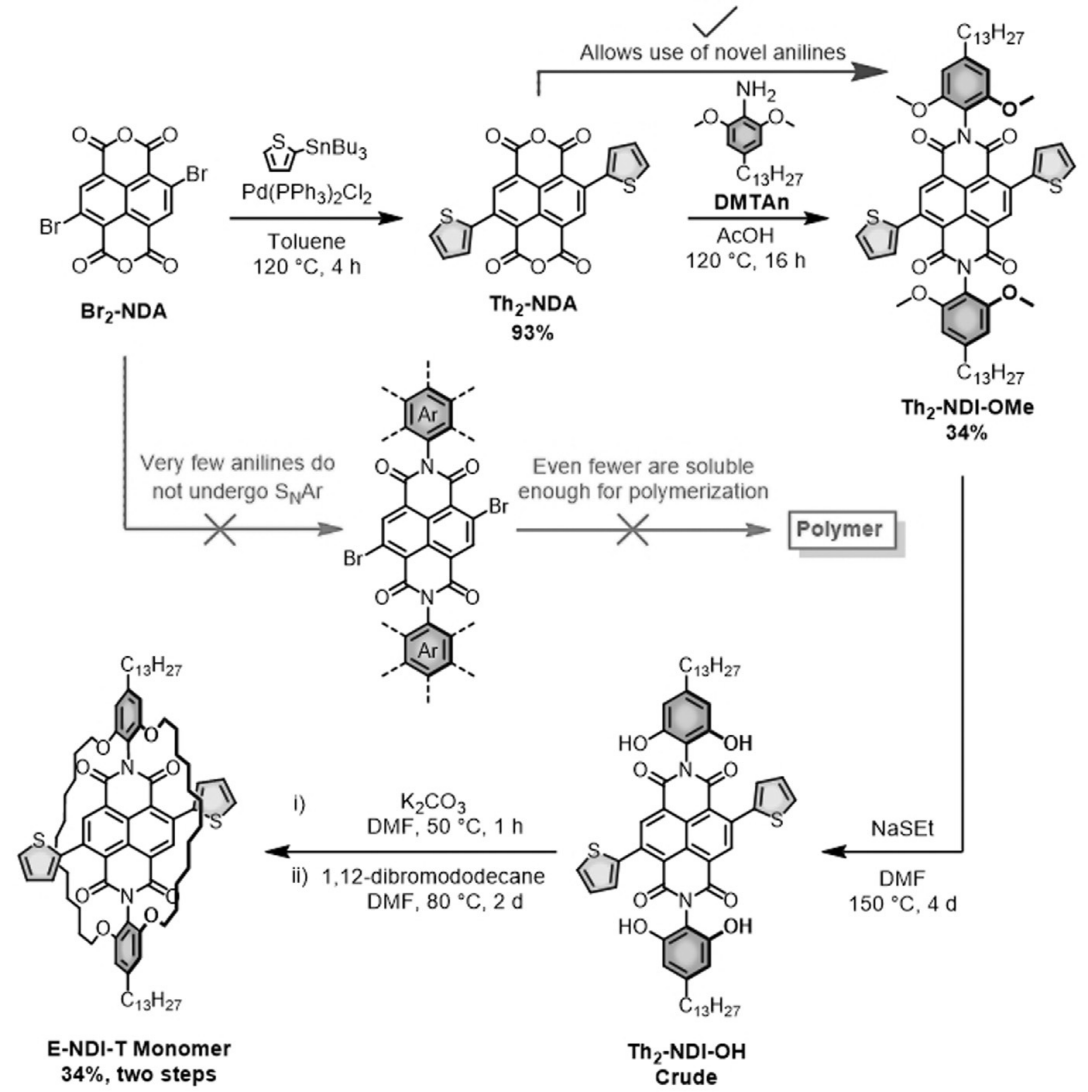
Synthesis of the encapsulated NDI monomer (**E‐NDI‐T**).

The preparation of the encapsulated NDI monomer (**E‐NDI‐T**) commenced with the synthesis of **Br_2_‐NDA** (Figure 1). **Br_2_‐NDA** can be obtained by brominating 1,4,5,8‐naphthalenetetracarboxylic dianhydride (NDA) using dibromoisocyanuric acid (DBI),[^23^, ^24^] bromine and catalytic iodine[^25^, ^26^] or 1,3‐dibromo‐5,5‐dimethylhydantoin (DBH)[^27^, ^28^] in oleum or concentrated sulfuric acid. In our hands, the use of DBH in sulfuric acid was the most reliable, leading to a mixture of brominated products (44 % yield); of which the 2,6‐isomer (**Br_2_‐NDA**) was the predominant product. Next, **Br_2_‐NDA** was treated with 2,6‐dimethoxy‐4‐tridecylaniline (**DMTAn**) in an attempt to prepare **Br_2_‐NDI‐OMe**. However, despite the use of acidic conditions, **DMTAn** did not attack the carbonyls of the anhydride. Instead, nucleophilic aromatic substitution (S_N_Ar) occurred, thereby displacing the bromines with **DMTAn**. This was quite surprising considering that similar anilines, such as 2,6‐diisopropylaniline,^[29]^ 2,4,6‐trimethylaniline^[30]^ and a few other *para*‐functionalized anilines[^31^, ^32^, ^33^] had been used successfully before. Therefore, unless the oxygen lone pairs (on the methoxy groups) make **DMTAn** bulkier than 2,6‐diisopropylaniline, steric encumbrance is likely not the underlying reason. Instead, we suggest that the electronics of **DMTAn** disfavor nucleophilic attack at the anhydride carbonyl(s) via either hydrogen‐bonding interactions or inductive withdrawal from the methoxy groups. Therefore, in this study we propose an alternative strategy that should allow access to a larger variety of phenyl‐substituted NDI molecules by avoiding potential S_N_Ar pathways.

Here, **Br_2_‐NDA** was first converted into **Th_2_‐NDA** through a traditional Stille cross‐coupling reaction and subsequently functionalized with a more versatile aniline, such as **DMTAn** (Figure 1). Our methodology therefore allows for a much wider use of solubilizing/functional groups at the imide nitrogen positions, which would not be possible via the traditional route (indicated in the middle). **Th_2_‐NDA** was obtained in 93 % using commercial **Br_2_‐NDA** or 39 % from the “home‐made” mixture of brominated products. The presence of other, unwanted brominated products in the “home‐made” Br2‐NDA sample therefore affords a lower yield upon isolation/purification. Next, **Th_2_‐NDA** was reacted with **DMTAn**, leading to the formation of **Th_2_‐NDI‐OMe** in 34 % yield. Subsequent treatment of **Th_2_‐NDI‐OMe** with excess sodium ethane thiolate successfully generated the crude demethylated product (**Th_2_‐NDI‐OH**), which was encapsulated with 1,12‐dibromododecane to give the encapsulated NDI monomer (**E‐NDI‐T**) in a two‐step yield of 34 %.

**Figure 2:**
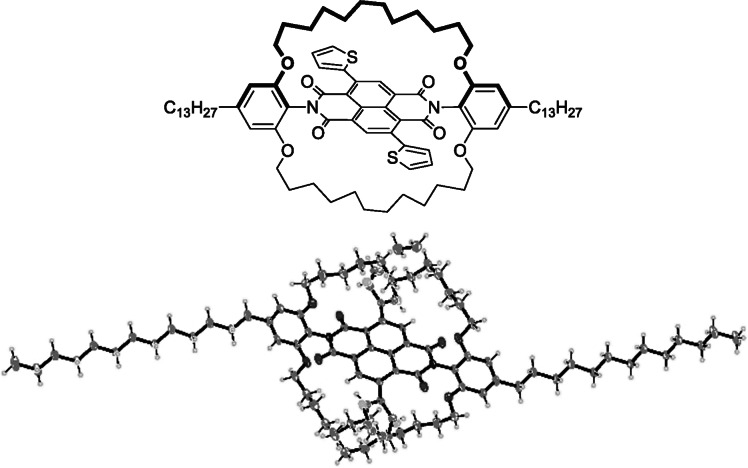
(Top) Chemical structure of **E‐NDI‐T**. (Bottom) Crystal structure of **E‐NDI‐T** (CCDC number: 2079163), including structural disorder in the thiophene rings and encapsulating straps.^[38]^

The structure of the encapsulated NDI monomer; **E‐NDI‐T**, was determined by X‐ray crystallography (single crystals grown via the layering technique using chloroform and methanol), which confirmed that the straps indeed shield the NDI core (Figure 2). Analysis of the packing shows minimum distances of ≈12 Å between the centroids of the NDI cores (see Supporting Information), with the closest intermolecular separations being between the alkyl chains, of the order of 4 Å. Analysis of the packing shows minimum distances of ≈12 Å between the centroids of the NDI cores (see Supporting Information), with the closest intermolecular separations being between the alkyl chains, of the order of 4 Å. In contrast, non‐encapsulated NDIs often show close π‐π stacking distances in the region of ≈3.3 Å,^[34]^ demonstrating the ability of the macrocycle to spatially separate the chromophores effectively.

The molecule is centrosymmetric in the crystal structure, with dihedral angles of ≈40° between the NDI and thiophene units. This is consistent with other reports on NDI‐T based polymers, which indicate a large degree of non‐coplanarity between the thiophene and the naphthalene core.[^35^, ^36^, ^37^]

**Figure 3:**
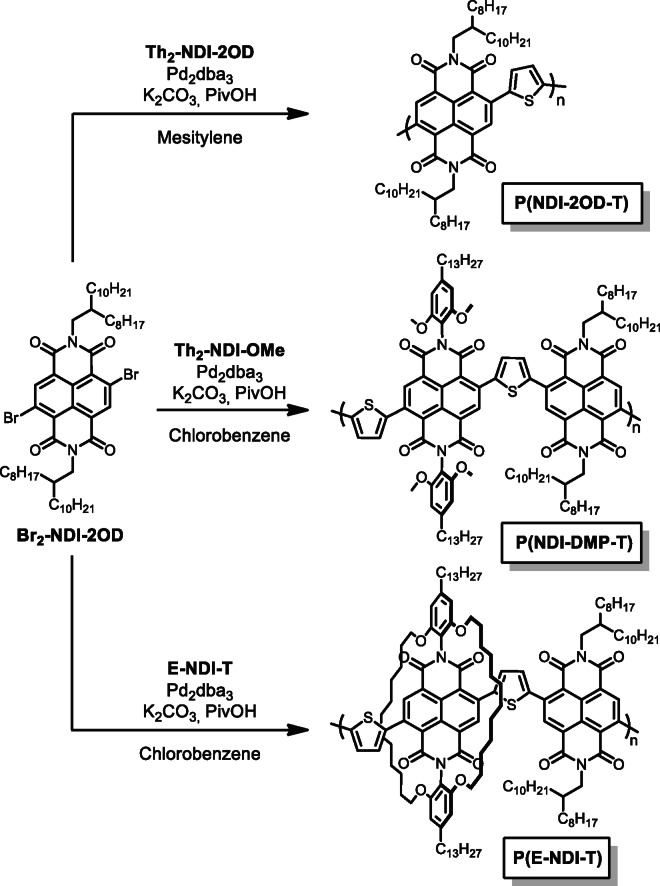
Synthesis of **P(NDI‐2OD‐T)**, **P(NDI‐DMP‐T)**, and **P(E‐NDI‐T)** using direct arylation polymerization (DArP).

The conjugated polymers (Figure 3) were prepared through a direct arylation polymerization (DArP) procedure, using *N*,*N*′‐bis(2‐octyldodecyl)‐2,6‐dibromo‐1,4,5,8‐naphthalene diimide (**Br_2_‐NDI‐2OD**) as the co‐monomer. DArP was chosen as the preferred method for this study since it is an emerging, green approach that enables the direct coupling of aryl halides (Ar‐X) and (hetero)aryls (Ar‐H), while avoiding toxic side products.[^39^, ^40^, ^41^, ^42^] Furthermore, recent studies have demonstrated that polymers prepared via DArP can achieve very similar molecular masses (M_n_ and M_w_) and device performances compared to traditional methods.[^39^, ^42^] As observed, the polymer series consists of the reference polymer **P(NDI‐2OD‐T)**, an in‐between, bulky dimethoxyphenyl polymer **P(NDI‐DMP‐T)**, and lastly the encapsulated **P(E‐NDI‐T)** polymer. All polymers were obtained in similar number average molecular weights (M_n_) of approximately 15 kDa (Table 1) and display good solubility in various solvents.

**Table 1:** Physical properties of the conjugated NDI polymers. The HOMO energy levels were measured using PESA.

Polymer	M_n_ [kg mol^−1^]	M_w_ [kg mol^−1^]	Dispersity	HOMO [eV]
**P(NDI‐2OD‐T)**	16.1	42.2	2.62	−5.8
**P(NDI‐DMP‐T)**	16.3	29.5	1.81	−5.75
**P(E‐NDI‐T)**	14.6	38.4	2.46	−5.75

### Optical Properties

**Figure 4:**
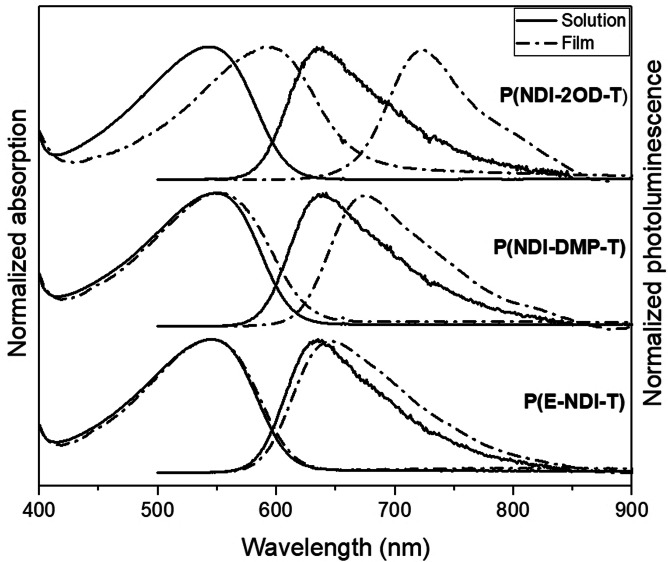
Normalized absorption and photoluminescence spectra of P(NDI‐2OD‐T) (top), P(NDI‐DMP‐T) (middle) and P(E‐NDI‐T) (bottom) in a 0.01 g L^−1^ chloroform solution (solid line) and ≈100 nm thin film on spectrosil substrates, not corrected for reflectivity (dash‐dot line).

The solution and thin film absorption and emission spectra of the three NDI polymers are shown in Figure 4 and summarized in Table 3. As observed, the spectral profiles of the solution absorption spectra are relatively featureless and nearly identical for all polymers, having *λ*_max_ values of 545 nm, 549 nm, and 546 nm for **P(NDI‐2OD‐T)**, **P(NDI‐DMP‐T)** and **P(E‐NDI‐T)**, respectively. The thin film absorption spectra, on the other hand, show a much clearer difference between the different polymers. For **P(NDI‐2OD‐T)**, *λ*_max_ is significantly red‐shifted (by 51 nm) to 596 nm on going from solution to solid state. In contrast, **P(NDI‐DMP‐T)** and **P(E‐NDI‐T)** display only minor redshifts of 3 and 1 nm, respectively. In other words, with increased shielding of the polymer backbone, the formation of lower energy species is increasingly suppressed, with a concomitant retention of the absorption profile when going from solution to thin film. This trend is consistent with previous studies on encapsulated conjugated materials.[^13^, ^14^, ^43^]

Similarly, the photoluminescence (PL) of the three polymers in solution is almost identical, with a *λ*_max_ at ≈636 nm. This suggests that, in solution, the emission originates from isolated chains. In thin film, on the other hand, the spectra are again progressively red‐shifted according to the “exposure” of the polymer chains to one another. For **P(NDI‐2OD‐T)**, the *λ*_max_ red‐shifts from 636 nm to 725 nm when going from solution to solid state. For **P(NDI‐DMP‐T)** instead, the PL maximum is only red‐shifted by 31 nm (from *λ*_max_=639 nm to 670 nm) in film compared to solution, thus indicating some remnant interstrand interaction. The discrepancy with the absorption results, which hint instead at a more significant suppression of interpolymer interactions, confirms the higher sensitivity of luminescence vs. absorption spectroscopy. Importantly however, the **P(E‐NDI‐T)** PL spectrum remains essentially identical when going from solution to the solid state, with the *λ*_max_ only red‐shifted by 5 nm (from 635 nm to 640 nm). This observation confirms effective suppression of solid‐state interactions and thus lower energy states in **P(E‐NDI‐T)**.

### Film Microstructure

**Figure 5:**
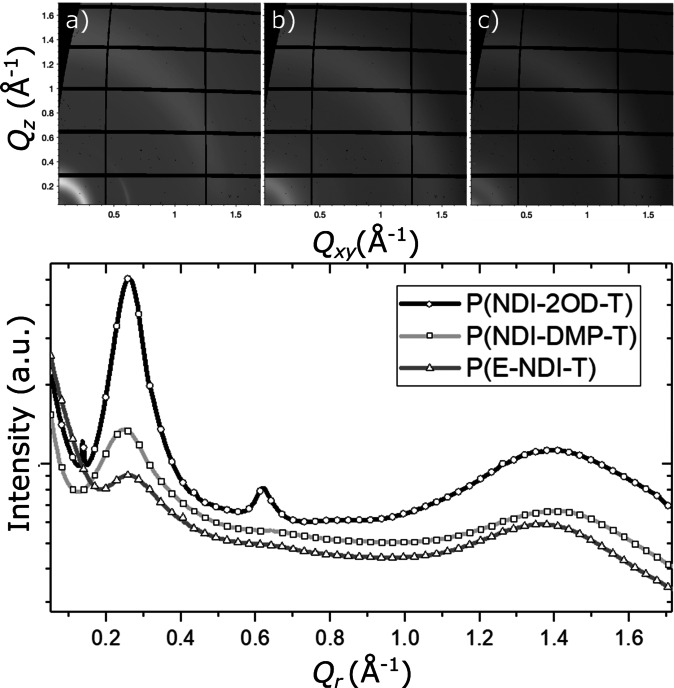
GIWAXS scattering data of a) **P(NDI‐2OD‐T)**, b) **P(NDI‐DMP‐T)**, and c) **P(E‐NDI‐T)**, and their respective radial integrations (below).

Grazing incidence wide‐angle X‐ray scattering (GIWAXS) was performed on all three polymers to elucidate the origins of the observations from the optical spectroscopy. Figure 5 shows the two‐dimensional scattering data and the corresponding radial integrals, with Table 2 containing the extracted d‐spacings of the novel polymeric materials.

**Table 2:** Summary of the extracted d‐spacings in **P(NDI‐2OD‐T)**, **P(NDI‐DMP‐T)** and **P(E‐NDI‐T)**.

	Å^−1^			Å		
	100	001	010	100	001	010
**P(NDI‐2OD‐T)**	0.26	0.62	1.39	24.169	10.121	4.51
**P(NDI‐DMP‐T)**	0.25	NA	1.37	25.316	NA	4.59
**P(E‐NDI‐T)**	0.26	NA	1.33	24.169	NA	4.72

The GIWAXS data for the parent polymer **P(NDI‐2OD‐T)** exhibits three prominent scattering features, a Debye‐Sherrer‐ring at 0.26 Å^−1^, a partial ring scattering ring at 0.62 Å^−1^, and a Debye‐Sherrer‐ring at 1.4 Å^−1^. These features are consistent with previous reports of closely related polymers,[^5^, ^44^, ^45^, ^46^] and as‐such, the observed scattering features may be ascribed to typical 100 lamellar packing with a distance of 25 Å (*Q*=0.26 Å^−1^) and 010 interchain stacking with a separation of 4.5 Å (*Q*=1.39 Å^−1^). The second prominent scattering feature at 0.62 Å^−1^ is not consistent with a higher order n00 reflection, however, the lengthscale of this feature 10.1 Å correlates well with the chain backbone repeat distance, consistent with the findings of Rivnay et al.^[44]^ The isotropic 100 and 010 reflections indicate that the film comprises randomly orientated crystalline grains with no preferential ordering either in‐ or out‐of‐plane.

On addition of the dimethoxyphenyl group in **P(NDI‐DMP‐T)**, the lamella scattering feature becomes less prominent and is further reduced for the fully encapsulated polymer **P(E‐NDI‐T)**, indicating a significant reduction in the long‐range order of these systems. Whilst the 010 feature is broad, it can clearly be seen that by going from the parent polymer **P(NDI‐2OD‐T)** to the non‐encapsulated **P(NDI‐DMP‐T)** and finally **P(E‐NDI‐T)**, the stacking distance along the π‐conjugated plane increases and becomes more diffuse, as expected from the structural changes. Therefore, with the combined photophysical and crystallographic characterization we can report with confidence that in the solid state, we are systematically increasing the distance and thus reducing the interactions between adjacent polymer chains.

### Photoluminescence

To further investigate the effects of the interchromophore interactions, the PLQYs of the three different polymers in both solution and solid state were also measured. The results are summarized in Table 3. As observed, the solution PLQYs of all polymers are relatively low, with 3.6 %, 4.1 % and 3.2 % for **P(NDI‐2OD‐T)**, **P(NDI‐DMP‐T)** and **P(E‐NDI‐T)**, respectively. However, surprisingly, the thin film PLQYs for both **P(NDI‐2OD‐T)** and **P(NDI‐DMP‐T)** are enhanced considerably, reaching 9.2 % and 6.1 %, respectively, while **P(E‐NDI‐T)** only exhibits a small increase, going from 3.2 to 3.7 %. The increase in PLQY for both **P(NDI‐2OD‐T)** and **P(NDI‐DMP‐T)** can therefore be associated with aggregation‐induced enhanced emission (AIEE), whereas **P(E‐NDI‐T)** mostly suppresses intermolecular interactions and hence retains its PLQY at higher concentrations (i.e., in thin films).

**Table 3:** Summary of the photophysical properties of **P(NDI‐2OD‐T)**, **P(NDI‐DMP‐T)** and **P(E‐NDI‐T)**.

Sample	*λ*_max,abs_ [nm]	*λ*_max,em_ [nm]	*φ* [%]^[a]^	PL lifetimes [ns]^[b]^			Average PL lifetime [ns]	*k*_r_^[c]^ [×10^8^ s^−1^]	*k*_nr_^[c]^ [×10^8^ s^−1^]
**P(NDI‐2OD‐T)** in CHCl_3_	545	636	3.6±0.3	*τ*_1_=0.31 (97.0 %)		*τ*_3_=4.26 (3.0 %)	*τ*_AVG_=0.429	0.84	22.47
**P(NDI‐DMP‐T)** in CHCl_3_	549	639	4.1±0.1	*τ*_1_=0.29 (78.2 %)	*τ*_2_=0.61 (19.6 %)	*τ*_3_=5.60 (2.2 %)	*τ*_AVG_=0.470	0.87	20.40
**P(E‐NDI‐T)** in CHCl_3_	546	635	3.2±0.2	*τ*_1_=0.27 (72.2 %)	*τ*_2_=0.57 (25.4 %)	*τ*_3_=4.36 (2.4 %)	*τ*_AVG_=0.444	0.72	21.80
**P(NDI‐2OD‐T)** film	596	725	9.2±0.3	*τ*_1_=0.89 (92.1 %)		*τ*_3_=4.26 (7.9 %)	*τ*_AVG_=1.156	0.80	7.86
**P(NDI‐DMP‐T)** film	552	670	6.1±0.3	*τ*_1_=0.23 (35.5 %)	*τ*_2_=0.61 (61.0 %)	*τ*_3_=5.60 (3.5 %)	*τ*_AVG_=0.65	0.94	14.45
**P(E‐NDI‐T)** film	547	640	3.7±0.2	*τ*_1_=0.20 (63.1 %)	*τ*_2_=0.57 (34.0 %)	*τ*_3_=4.36 (2.9 %)	*τ*_AVG_=0.446	0.83	21.59

[a] Excitation at 520 nm, [b] Fluorescence lifetimes measured at 375 nm, and percentage weights are reported between parentheses. [c] Rate constants for radiative (*k*_r_, 10^8^ s^−1^) and non‐radiative decay (*k*_nr_, 10^8^ s^−1^) were calculated from the *φ* and *τ* values according to the formulae *k*_r_=*φ*/*τ* and *k*_nr_=(1−*φ*)/*τ*, where *τ* corresponds to the weighted average of the lifetimes.

The PL lifetimes and rate constants for radiative (*k*_r_) and non‐radiative (*k*_nr_) decay provide further insight (Table 3). In line with the PLQYs, for all polymers the weighted‐average PL lifetimes (*τ*_AVG_) are very similar in solution, yielding comparable values of *k*_r_ and *k*_nr_. Combined with the near‐indistinguishable profiles of their solution PL spectra, it can be concluded that the PL properties of the isolated chains of **P(NDI‐2OD‐T)**, **P(NDI‐DMP‐T)** and **P(E‐NDI‐T)** are very similar.

For all polymers, the values of the average lifetime *τ*_AVG_ values are lower in solution than in thin films. For **P(NDI‐2OD‐T)**, both solutions and thin films follow a similar bi‐exponential decay, in which the fast‐decaying component (*τ*_1_) dominates exciton deactivation. As seen, the PL lifetime increases from *τ*_1_=0.31 ns (or *τ*_AVG_=0.43 ns) in solution to 0.89 ns (or *τ*_AVG_=1.16 ns) in film. This is manifested in a near three‐fold decrease in *k*_nr_ in films of **P(NDI‐2OD‐T)** (*k*_nr_=7.86×10^8^ s^−1^) compared to the solution value (*k*_nr_=22.47×10^8^ s^−1^). In contrast, *k*_r_ is effectively unchanged on going to the solid state. The absence of any additional fast‐decaying PL components in thin film, and the near identical values of *k*_r_ for **P(NDI‐2OD‐T)** in solution and thin film suggest that J‐aggregation is likely not responsible for its enhanced PL in the solid state.[^47^, ^48^]

For **P(NDI‐DMP‐T)**, a similar trend is observed. However, the difference between solution and thin films is much smaller due to partial suppression of interchromophore interactions. The average lifetimes for **P(NDI‐DMP‐T)** are 0.47 ns in solution and 0.65 ns in thin film, again closely matching the difference in PLQYs (4.1 % in solution vs. 6.1 % in thin film). This is similarly due to a decrease in *k*_nr_ in thin film compared to solution, albeit a smaller one than for **P(NDI‐2OD‐T)** (14.45×10^8^ s^−1^ in thin film vs. 20.40×10^8^ s^−1^ in solution). There is again no significant change in *k*_r_. In contrast, when going from solution to thin films the PL characteristics of **P(E‐NDI‐T)** remain relatively unaffected due to the suppression of intermolecular interactions by molecular encapsulation. This translates to a *τ*_AVG_ of 0.44 ns in solution and 0.45 ns in thin film, which is comparable to the small difference in PLQYs (3.2 % in solution versus 3.7 % in thin film). Hence, *k*_r_ and *k*_nr_ are effectively unchanged.

To summarize, as the interactions between polymer chains are increased in the order **P(E‐NDI‐T)** → **P(NDI‐DMP‐T)** → **P(NDI‐2OD‐T)** an enhancement of solid‐state PL is observed due to an improved suppression of non‐radiative decay pathways. The combined photophysical and crystallographic characterisation clearly shows that the substantial decrease in non‐radiative decay for **P(NDI‐2OD‐T)** in thin film is a consequence of stronger intermolecular interactions between polymer chains.

## Discussion

It is commonly assumed and observed that conjugated polymers undergo substantial photoluminescence quenching on going from solution to the solid state. Our combined morphological and photophysical results show the surprising result that i) the extremely well‐known polymer **P(NDI‐2OD‐T)** undergoes strong aggregation induced enhanced emission and ii) this phenomenon originates from the interchain packing. We (and others) have previously shown that molecular encapsulation allows a polymer to retain, to some extent, solution properties when in the solid state.[^13^, ^14^] In this work, we find the same concept applies to **P(E‐NDI‐T)** as both the absorption and emission shapes, yields and lifetimes are almost identical. However, in contrast to almost all other reported conjugated polymers, the emissivity of the non‐encapsulated derivative increases in the solid state (as opposed to decreasing), resulting in a relatively high emission PLQY for this wavelength region. The phenomenon of aggregation induced enhanced emission is well known and has been reported in numerous conjugated polymer systems, including those containing naphthalene diimide units.^[49]^ Crucially however, in virtually every example of conjugated polymers AIEE‐specific groups (such as vinylenes or TPEs), that are well known to imbue AIEE‐like properties, are introduced for this reason.[^50^, ^51^, ^52^, ^53^, ^54^, ^55^] There are almost no examples of a “conventional” conjugated polymer undergoing aggregation induced enhanced emission. Moreover, it is remarkable that it has not been previously observed in **P(NDI‐2OD‐T)**, which have been extensively studied. In fact, we can find only one other example of AIEE in a conventional (i.e., without the inclusion of AIEE‐specific groups) conjugated polymer, where emissive efficiency was enhanced through the formation of close contacts between adjacent polymer chains.^[56]^

To determine the origin of the observed AIEE in this system it is useful to consider all the possible mechanisms through which it could be occurring. NDI‐based small molecules have previously been shown to undergo enhanced emission in the solid state predominantly through J‐aggregation.[^57^, ^58^, ^59^, ^60^, ^61^] However, the π‐stacking distance as measured by GIWAXS for **P(NDI‐2OD‐T)** is ≈4.5 Å, which is considerably longer than those reported for small molecule NDIs displaying J‐aggregates (<4 Å). We also do not observe a sharpening of the absorption spectrum or an enhancement in *k*_r_ in the solid state, which are common spectroscopic signatures of J‐aggregation. Furthermore, the red‐shifted emission of NDI‐T based polymers has been demonstrated to originate from the crystallization induced planarization of the conjugated backbone rather than J‐aggregates.^[62]^ We observe a stepwise reduction in AIEE when going from **P(NDI‐2OD‐T)** to **P(NDI‐DMP‐T)** and **P(E‐NDI‐T)** films, which correlates extremely well with the increased 010 distances interlayer separation. Thus, the crystallization of the π‐conjugated faces of the polymer results not only in a planarization, but also in the suppression of non‐radiative decay pathways, and thereby a substantially increased photo‐luminescence efficiency. We believe that this is the first observation of this effect in a non‐vinylene containing conjugated polymer and suggest that it could provide a novel design strategy for obtaining good emissive efficiency in narrow band gap conjugated polymers—a major hurdle that must be overcome to increase the efficiency of OPV devices.^[10]^ Finally, we note that **P(NDI‐2OD‐T)** and related polymers are the most successful conjugated polymers used in all‐polymer solar cells^[63]^ and this previously unreported AIEE may provide a clue as to why they have performed so well.

## Conclusion

This article reports the synthesis of molecularly encapsulated, naphthalene diimide based conjugated polymers through the development of new synthetic pathways. The encapsulated polymer allows us to determine the exact effect of interpolymer interactions on the optical properties of the conjugated polymer. Surprisingly, in the solid state, the encapsulated polymer has a higher energy, but lower photoluminescence quantum yield compared to the naked reference polymer, which is in contrast to the current conventional wisdom and a violation of the energy gap “law”.[^64^, ^65^] Instead, this work reveals a previously unrecognized aggregation induced enhanced emission phenomena in one of the most well studied conjugated polymers **P(NDI‐2OD‐T)**. Structural characterization confirms that this AIEE is due to the crystallization of the π‐conjugated faces of the polymer chains. Therefore, we believe that understanding and having control over the crystallization and solid‐state behavior of conjugated polymers may be key to enhance their emissive properties. Our results conclusively demonstrate that solid state photoluminescence in conjugated polymers can actually be enhanced through closer packing (which is often beneficial for charge transport) suggesting that high PLQY and high charge carrier mobility may not be as mutually exclusive as is commonly believed.

## Conflict of interest

The authors declare no conflict of interest.

## Supporting information

As a service to our authors and readers, this journal provides supporting information supplied by the authors. Such materials are peer reviewed and may be re‐organized for online delivery, but are not copy‐edited or typeset. Technical support issues arising from supporting information (other than missing files) should be addressed to the authors.

Supporting Information
