# Supplementary material for: Molecular Encapsulation of Naphthalene Diimide (NDI) Based π‐Conjugated Polymers: A Tool for Understanding Photoluminescence
Source: Angew Chem Int Ed Engl. 2021 Oct 18;60(47):25005–12. doi: 10.1002/anie.202110139 (PMC9297952; doi:10.1002/anie.202110139)
Supplement: Supplementary file 1 — Supporting Information [file ANIE-60-25005-s001.pdf]

Angewandte Chemie
Eine Zeitschrift der Gesellschaft Deutscher Chemiker

Supporting Information

Molecular Encapsulation of Naphthalene Diimide (NDI) Based π-Conjugated Polymers: A Tool for Understanding Photoluminescence

*Jeroen Royakkers[+], Kunping Guo[+], Daniel T. W. Toolan, Liang-Wen Feng, Alessandro Minotto, Daniel G. Congrave, Magda Danowska, Weixuan Zeng, Andrew D. Bond, Mohammed Al-Hashimi, Tobin J. Marks, Antonio Facchetti, Franco Cacialli, and Hugo Bronstein**

anie_202110139_sm_miscellaneous_information.pdf

Table of Contents

General Experimental Information 2

Synthesis 3

NMR Spectra 11

GPC Traces 22

HOMO and LUMO Distributions 23

TD-DFT 25

AIEE Study 26

Transient Photoluminescence Lifetimes 28

Grazing Incidence X-ray Scattering studies 31

OPV Device Data 31

References 33

General Experimental Information

All reactions were performed in dry glassware under argon or nitrogen atmosphere and magnetic stirring, unless stated otherwise. Light-sensitive reactions were protected using aluminum foil. Chemicals and (anhydrous) solvents were purchased from chemical suppliers (Sigma-Aldrich, TCI, Fluorochem, Acros Organics, Alfa Aesar, SLS, Fisher Scientific) and used as received unless stated otherwise. Reactions were monitored through thin layer chromatography (TLC) using DC Fertigfolien ALUGRAM aluminium sheets coated with silica gel. Column chromatography was carried out using Geduran silica gel 60 (40-63 µm) or Biotage® Isolera™ Four with Biotage® SNAP/ SNAP ultra-cartridges (10 g, 20 g, 50 g or 100 g). ^{1}H NMR spectra were recorded on a 400 MHz Avance III HD Spectrometer, 400 MHz Neo Prodigy Spectrometer, 400 MHz Smart Probe Spectrometer or a 500 MHz DCH Cryoprobe Spectrometer in the stated solvent using residual protic solvent $CHCl_3$ (δ = 7.26 ppm, s) or DMSO (δ = 2.50 ppm, s) as the internal standard. ^{1}H NMR chemical shifts are reported to the nearest 0.01 ppm. ^{13}C NMR spectra were recorded on the 500 MHz DCH Cryoprobe Spectrometer in the stated solvent using the residual protic solvent $CHCl_3$ (δ= 77.16 ppm, t) or DMSO (δ = 39.52 ppm, s) as the internal standard. ^{13}C NMR chemical shifts are reported to the nearest 0.01 ppm. Mass spectra were obtained using a Waters LCT Premier, Waters Vion IMS Qtof, Finnigan MAT 900XP or Waters MALDI micro MX spectrometer at the Department of Chemistry, University of Cambridge. The number-average (Mn) and weight-average (Mw) molecular weights were determined against a polystyrene standard using an Agilent Technologies 1200 series GPC in chlorobenzene at 80 °C.

The samples for the photophysical measurements were prepared by dissolving the polymers in spectroscopic grade chloroform (over 12 hours in a glovebox under N2 atmosphere). The concentration of the solutions was 10 mg/mL. The films were spin-coated under N2 (at 800 rpm) onto fused silica substrates using the 10 mg/mL chloroform solutions. The thickness of the obtained films was ≈ 100 nm, as measured with a Dektak profilometer. The absorption spectra were measured by using an Agilent 8453 UV-Vis spectrometer. Photoluminescence (PL) spectra were collected with an Andor Shamrock SR-163 spectrograph (with the Czerny-Turner optical layout, a 300 lines/mm 500 nm blazed grating, a 163 mm focal length, and an f/3.6 numerical aperture) coupled to a silicon-based Andor Newton electron multiplying charge-coupled device with a maximum resolution of 0.77 nm. All PL spectra were corrected for the spectral responsivity of the instrument. The PL efficiency characterization was carried out using a 520 nm diode laser (Thorlabs) and an integrating sphere setup (Bentham), following the procedure reported in De Mello et al. in 1997.[1] All absorption and PL spectra were collected in air at room temperature. Time-resolved fluorescence measurements were carried out with a time correlated single photon counting (TCSPC) spectrometer (Edinburgh instruments, LifeSpec II) by exciting the samples at 375 nm with a ps-pulsed diode laser (Edinburgh Instruments EPL 445). The PL lifetimes reported in Table 3 were extracted via global fitting of the PL decays measured at different emission wavelengths with an n-exponential function. In such a global analysis, the lifetimes at different

wavelengths are linked while the amplitudes of each exponential component can vary for each decay. Global Least Squares Analysis (GLSA) was carried out using the Edinburgh instruments Fluorescence Analysis Software Technology (FAST) software package.

Synthesis

Encapsulated Monomer synthesis

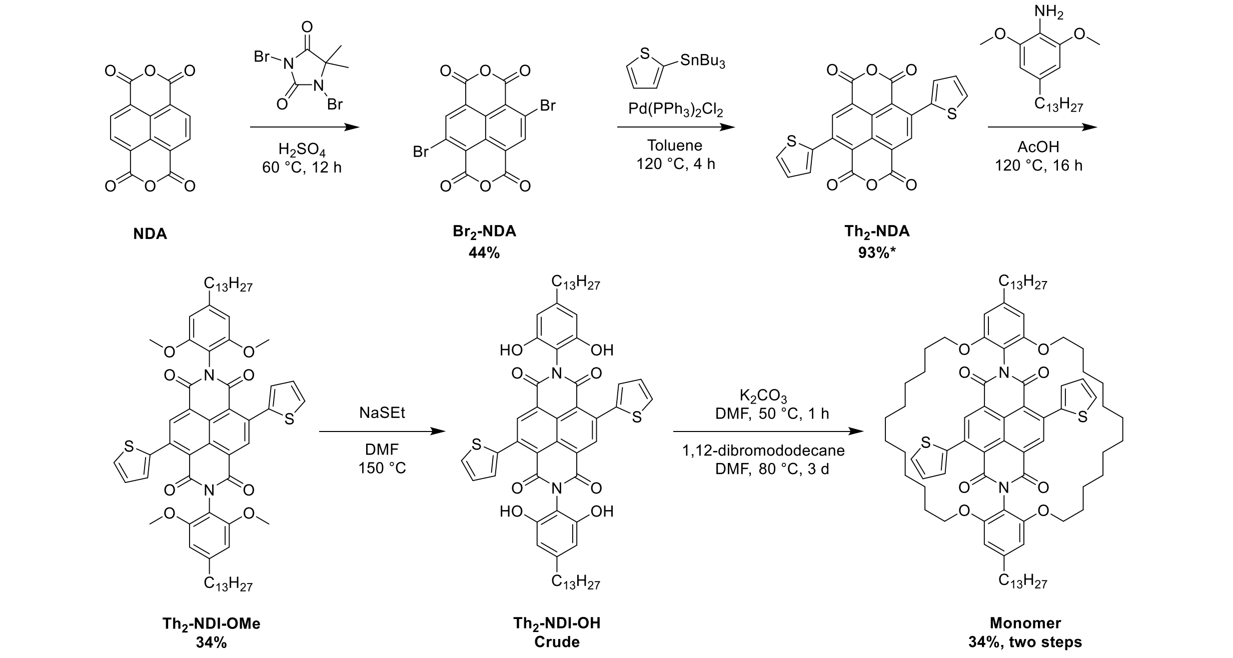

2,6-dibromo-1,4,5,8-naphthalenetetracarboxylic acid dianhydride (Br_2-NDA)[2–4]

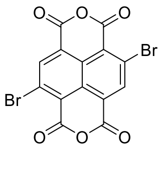

Under Argon, 1,3-dibromo-5,5-dimethylhydantoin (14.94 g, 52.22 mmol) was added portion wise (4 portions over 1 h) to a slurry of 1,4,5,8-Naphthalenetetracarboxylic dianhydride (10 g, 37.08 mmol) in sulphuric acid (100 mL) and the reaction was heated to 60 ^{o}C for 12 h. The resulting reaction mixture was poured carefully onto ice (500 mg) and H_2O (500 mL) was added. The mixture was stirred in ice water for half an hour. Next, the yellow solids were collected by filtration, sonicated in methanol and filtered again to yield a mixture of brominated products. The crude product was placed into a beaker together with hot DMF (~ 4 mL/g). At room temperature, the suspension was filtered, and the yellow solid product was washed with methanol. Lastly, the product was sonicated in methanol and collected by filtration to yield relatively pure Br_2-NDA (9.7875g, 22.9768 mmol, 44%).

^{1}H NMR (400 MHz, DMSO) δ 8.79 (s, 2H); **HRMS** (ASAP-TOF): Calculated for $C_{14}H_3O_6Br_2^+$: 424.8296. Found *m/z* 424.8299 $[M+H]^+$.

(2,6-thiophen-2-yl)naphthalene-1,4,5,8-tetracarboxylic Dianhydride (Th_2-NDA)[5]

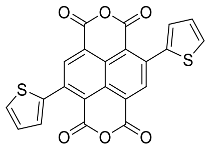

Under N_2, Br_2-NDA (880 mg, 2.0662 mmol) and $Pd(PPh_3)_2Cl_2$ (145.2 mg, 0.2069 mmol) were placed into a three-necked round-bottomed flask (equipped with reflux condenser). It was flushed with argon for 10 min. Next, anhydrous toluene (26 mL) and 2-(tributylstannyl)thiophene (1.76 mL, 5.5414 mmol) were added. The mixture was heated to 120 °C and left stirring for 8 h. The dark red solids were collected by filtration (while warm) and washed with toluene. Next, the solids were boiled and filtered from a minimal amount of methanol and then from chloroform. The desired product was obtained as a red solid (831.2 mg, 1.9222 mmol, 93%).

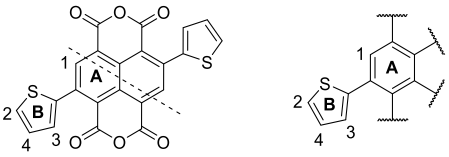

^{1}H NMR (400 MHz, DMSO) δ 8.50 (s, 2H, #1), 7.91 (dd, *J* = 5.1, 1.2 Hz, 2H, #2), 7.57 (dd, *J* = 3.5, 1.0 Hz, 2H, #3), 7.27 (dd, *J* = 5.0, 3.6 Hz, 2H, #4). **HRMS** (IMS-QTOF): Calculated for $C_{22}H_8O_6S_2^+$: 431.9762. Found *m*/*z* 431.9761 $[M]^-$.

2,7-bis(2,6-dimethoxy-4-tridecylphenyl)-4,9-di(thiophen-2-yl)benzo[lmn][3,8]phenanthroline-1,3,6,8(2H,7H)-tetraone (Th_2-NDI-OMe)

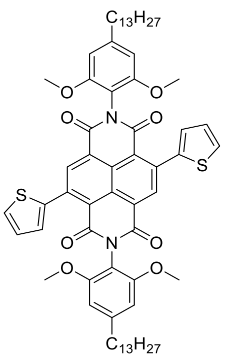

Under argon, 2,6-dimethoxy-4-tridecylaniline (620.73 mg, 1.85 mmol) and Th_2-NDA (200 mg, 0.4625 mmol) were dissolved in glacial acetic acid (12 mL) and heated to 120 °C. The reaction was left stirring for 3 h. The reaction mixture was added slowly into stirring MeOH (100-150 mL). After 10 minutes, the resulting orange solids were collected by filtration, dissolved in the minimal amount of a 1-to-1 mixture of DCM and hexane and wet-loaded onto a silica column. The crude was purified via silica column chromatography using chloroform/hexane and later chloroform/methanol (stepwise column from 50% $CHCl_3$ in hexane, to 100% $CHCl_3$ and finally 2% MeOH in $CHCl_3$). The product fractions

were concentrated *in vacuo,* sonicated in methanol and collected by filtration (wash with MeOH) to yield pure, bright orange solid product (167.5 mg, 0.1569 mmol, 34%)

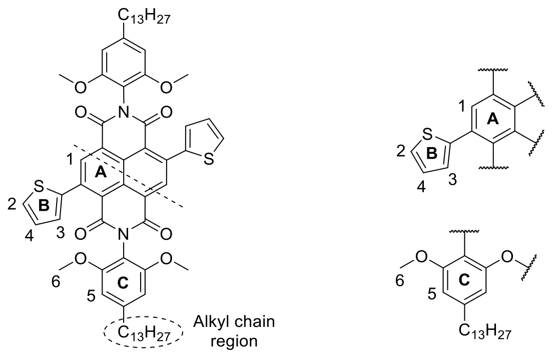

R$_f$ = 0.37 (100% $CHCl_3$); **^{1}H NMR** (500 MHz, $CDCl_3$) δ 8.81 (s, 2H, #1), 7.47 (dd, *J* = 5.1, 1.2 Hz, 2H, #2), 7.31 (dd, *J* = 3.6, 1.2 Hz, 2H, #3), 7.11 (dd, *J* = 5.1, 3.6 Hz, 2H, #4), 6.48 (s, 4H, #5), 3.75 (s, 12H, #6), 2.66 – 2.59 (m, 4H), 1.65 (dt, *J* = 15.7, 7.7 Hz, 4H), 1.27 (s, 40H), 0.89 (t, *J* = 7.0 Hz, 6H). **^{13}C NMR{H}** (126 MHz, $CDCl_3$) δ 161.79, 161.49, 155.57, 145.89, 140.87, 140.11, 136.69, 128.34, 127.80, 127.31, 126.02, 124.04, 109.75, 104.62, 55.95, 37.04, 31.94, 31.34, 29.71, 29.65, 29.58, 29.38, 22.71, 14.14. **HRMS** (ASAP-TOF): Calculated for $C_{64}H_{79}N_2O_8S_2^+$: 1067.5278. Found *m/z* 1067.5228 $[M+H]^+$.

Encapsulated Monomer (E-NDI-T)

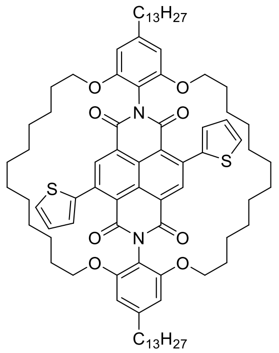

Step 1: Synthesis of crude Th_2-NDI-OH

To a 100 mL round-bottomed flask under argon, 2,7-bis(2,6-dimethoxy-4-tridecylphenyl)-4,9-di(thiophen-2-yl)benzo[lmn][3,8]phenanthroline-1,3,6,8(2H,7H)-tetraone (600 mg, 0.5621 mmol), NaSEt (technical ~90%, 1.4185 g, 16.8628 mmol) and anhydrous DMF (36 mL) were added and it was heated to 150 ºC for 4 days. The reaction mixture was poured into pre-stirring water (~200 mL) and 1M HCl (~50 mL) and filtered. The solids were dried *in vacuo*. The resulting crude NDI tetra-ol was obtained as a dark red solid (~568.5 mg) and used in the subsequent reaction without further purification.

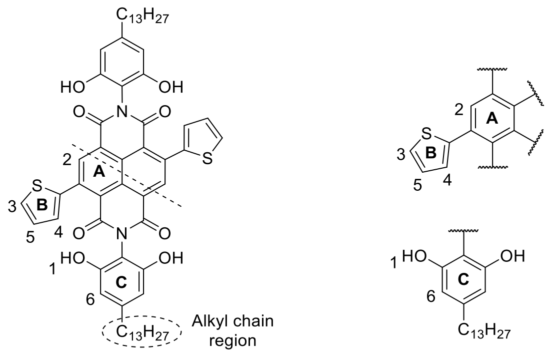

¹H NMR (400 MHz, DMSO) δ 9.33 (s, 4H, #1), 8.51 (s, 2H, #2), 7.78 (d, *J* = 4.7 Hz, 2H, #3), 7.39 (d, *J* = 2.9 Hz, 2H, #4), 7.20 – 7.13 (m, 2H, #5), 6.23 (s, 4H, #6), 2.44 (s, 4H), 1.55 (s, 4H), 1.25 (s, 40H), 0.85 (d, *J* = 6.8 Hz, 6H). **HRMS** (ASAP-TOF): Calculated for $C_{60}H_{71}N_2O_8S_2^+$: 1011.4674. Found *m*/*z* 1011.4690 $[M+H]^+$.

<u>Step 2:</u> Encapsulation of crude Th_2-NDI-OH

The crude Th_2-NDI-OH was placed into a 250 mL round-bottomed flask under argon. K_2CO_3 (413.3 mg, 2.9935 mmol) and anhydrous DMF (75 mL) were added and the reaction mixture was heated to 50 °C for 1 h. Next, a solution 1,12-dibromododecane (322.6 mg, 0.9832 mmol) in anhydrous DMF (75 mL) was added dropwise over 1 h and the mixture was heated to 80 °C for 2 days. The reaction mixture was concentrated *in vacuo*, dissolved in a minimal amount of DCM and hexane and purified by column chromatography (stepwise column; 50% $CHCl_3$ to 75-80% $CHCl_3$ and 100% $CHCl_3$ in hexane). The product fractions were concentrated *in vacuo* to afford red/orange coloured solid product (257.2 mg, 0.1913 mmol, 34% two-step yield).

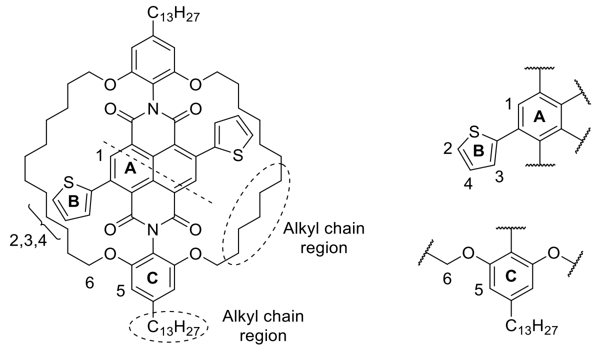

¹H NMR (500 MHz, $CDCl_3$) δ 8.83 (s, 2H, #1), 7.48 (dd, *J* = 5.1, 1.2 Hz, 2H, #2), 7.31 (dd, *J* = 3.6, 1.2 Hz, 2H, #3), 7.11 (dd, *J* = 5.1, 3.6 Hz, 2H, #4), 6.43 (s, 4H, #5), 3.96 (t, *J* = 5.3 Hz, 8H, #6), 2.60 – 2.55 (m, 4H), 1.63 – 1.57 (m, 10H), 1.26 (d, *J* = 6.3 Hz, 48H), 1.11 (dd, *J* = 16.0, 7.4 Hz, 8H), 0.90 – 0.85 (m, 14H), 0.73 – 0.67 (m, 6H). **¹³C NMR{H}** (126 MHz, $CDCl_3$) δ 161.62, 161.35, 155.31, 145.82, 140.87, 140.36, 136.81, 128.56, 128.28, 127.85, 127.35, 126.08, 123.98, 109.77, 105.12, 69.07, 37.14, 32.09, 31.44, 29.86, 29.85, 29.83, 29.81, 29.79, 29.73, 29.72, 29.53, 28.81, 28.72, 28.01, 27.82, 26.35, 22.86, 14.29. **HRMS** (IMS QTOF): Calculated for $C_{84}H_{114}N_2O_8S_2Na^+$: 1365.7909. Found *m*/*z* 1365.7896 $[M+Na]^+$. Structure confirmed by **X-ray crystallography**.

Reference Monomer Synthesis[10]

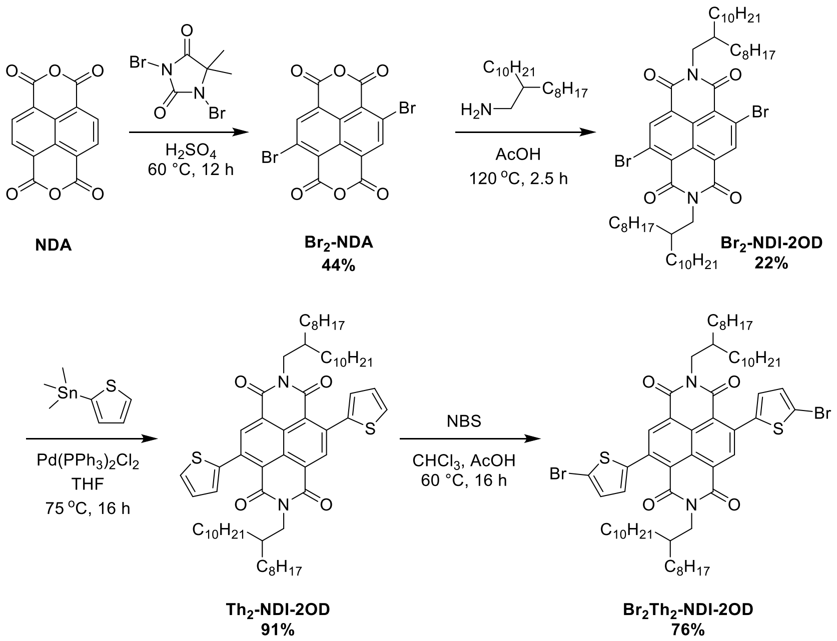

N,N′-bis(2-octyldodecyl)-2,6-dibromo-1,4,5,8-naphthalene diimide (Br_2-NDI-2OD)[10]

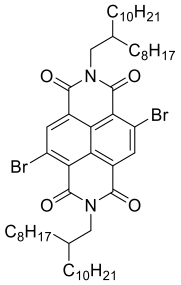

Under Argon to a single-necked 500 mL round-bottomed flask equipped with reflux condensor, Br_2-NDA (4.08 g, 9.5781 mmol) and 2-octyldodecylamine (9.85 g, 33.1013 mmol) were added and it was degassed for 10 min. Next, glacial acetic acid (200 mL) was added and it was heated to 120 ºC until all solids dissolved (~2.5 hours). The reaction mixture was purified using column chromatography (silica, chloroform) and the product fractions were concentrated *in vacuo*. Lastly, the solids were sonicated in acetone (3x) and collected by filtration to afford the product (2.0452 g, 2.0762 mmol, 22%).

^{1}H NMR (400 MHz, $CDCl_3$) δ 9.00 (s, 2H), 4.15 (d, J = 7.3 Hz, 4H), 1.99 (s, 2H), 1.41 – 1.17 (m, 64H), 0.89 – 0.83 (m, 12H). **HRMS** (IMS QTOF): Calculated for $C_{54}H_{84}Br_2N_2O_4^+$: 982.4798. Found m/z 982.4778 $[M]^+$.

N,N'-bis(2-octyldodecyl)-2,6-bis(thiophen-2-yl)-1,4,5,8-naphthalene diimide (Th_2-NDI-2OD)[6]

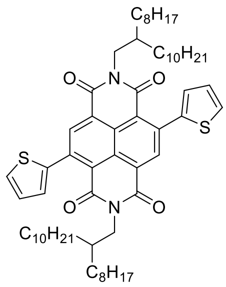

Under Argon, to a 25 mL microwave vial, NDI2OD-Br_2 (400 mg, 0.4061 mmol), $Pd(PPh_3)_2Cl_2$ (6.6 mg, 0.0093 mmol) were added and it was degassed for 10 min. Next, 2-(Tributylstannyl)thiophene (0.31 mL, 0.9783 mmol) and dry THF (9 mL) were added and it was heated at 75 ºC for 16 h. The reaction mixture was concentrated *in vacuo*, crystallized from the minimal amount of ^{i}PrOH and collected by filtration (wash with MeOH). The product was dried under high vacuum and obtained as an orange solid (366.1 mg, 0.3692 mmol, 91%).

^{1}H NMR (500 MHz, $CDCl_3$) δ 8.76 (s, 2H), 7.56 (dd, *J* = 5.1, 1.2 Hz, 2H), 7.29 (dd, *J* = 3.6, 1.2 Hz, 2H), 7.19 (dd, *J* = 5.1, 3.6 Hz, 2H), 4.06 (d, *J* = 7.4 Hz, 4H), 1.97 – 1.89 (m, 2H), 1.33 – 1.16 (m, 64H), 0.86 (q, *J* = 7.1 Hz, 12H). **^{13}C NMR{H}** (126 MHz, $CDCl_3$) δ 162.72, 162.52, 140.96, 140.38, 136.79, 128.36, 128.18, 127.61, 127.57, 125.54, 123.54, 45.04, 36.60, 32.06, 32.04, 31.72, 31.70, 30.20, 30.20, 29.81, 29.78, 29.76, 29.70, 29.49, 29.46, 26.53, 22.83, 22.81, 14.26. **HRMS** (IMS QTOF): Calculated for $C_{62}H_{90}N_2O_4S_2^{+}$: 990.6342. Found *m/z* 990.6322 $[M]^{+}$.

N,N'-bis(2-octyldodecyl)-2,6-bis(5-bromothiophen-2-yl)-1,4,5,8-naphthalene diimide (Br_2Th_2-NDI-2OD)[6]

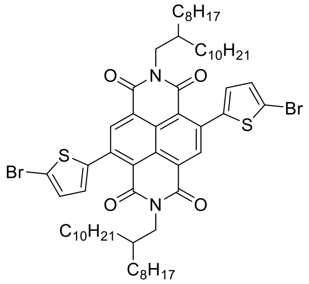

Under Argon, NDI2OD-Th_2 (365 mg, 0.3681 mmol) was dissolved in chloroform (11 mL) and AcOH (11 mL). To this, NBS (163.8 mg, 0.9203 mmol) was added in one portion and it was stirred for 16 h at 60 ºC. The orange solution turned dark red. The reaction mixture was concentrated in vacuo, sonicated in MeOH (2x) and collected by filtration. Next, the solids were boiled in ethanol and collected by filtration. Lastly, the solids were purified via silica column chromatography (1:1 DCM/Hexane) to afford the product as pure, dark red/purple solids (321.0 mg, 0.2793 mmol, 76%).

^{1}H NMR (400 MHz, $CDCl_3$) δ 8.72 (s, 2H), 7.14 (d, *J* = 3.7 Hz, 2H), 7.08 (d, *J* = 3.7 Hz, 2H), 4.07 (d, *J* = 7.2 Hz, 4H), 1.94 (s, 2H), 1.25 (d, *J* = 20.3 Hz, 64H), 0.86 (q, *J* = 6.3 Hz, 12H). **HRMS** (IMS QTOF): Calculated for $C_{82}H_{115}N_2O_8S_2{}^{79}Br^{81}Br^+$: 1171.4429 Found *m/z* 1171.4436 $[M+Na]^+$.

Polymer Synthesis

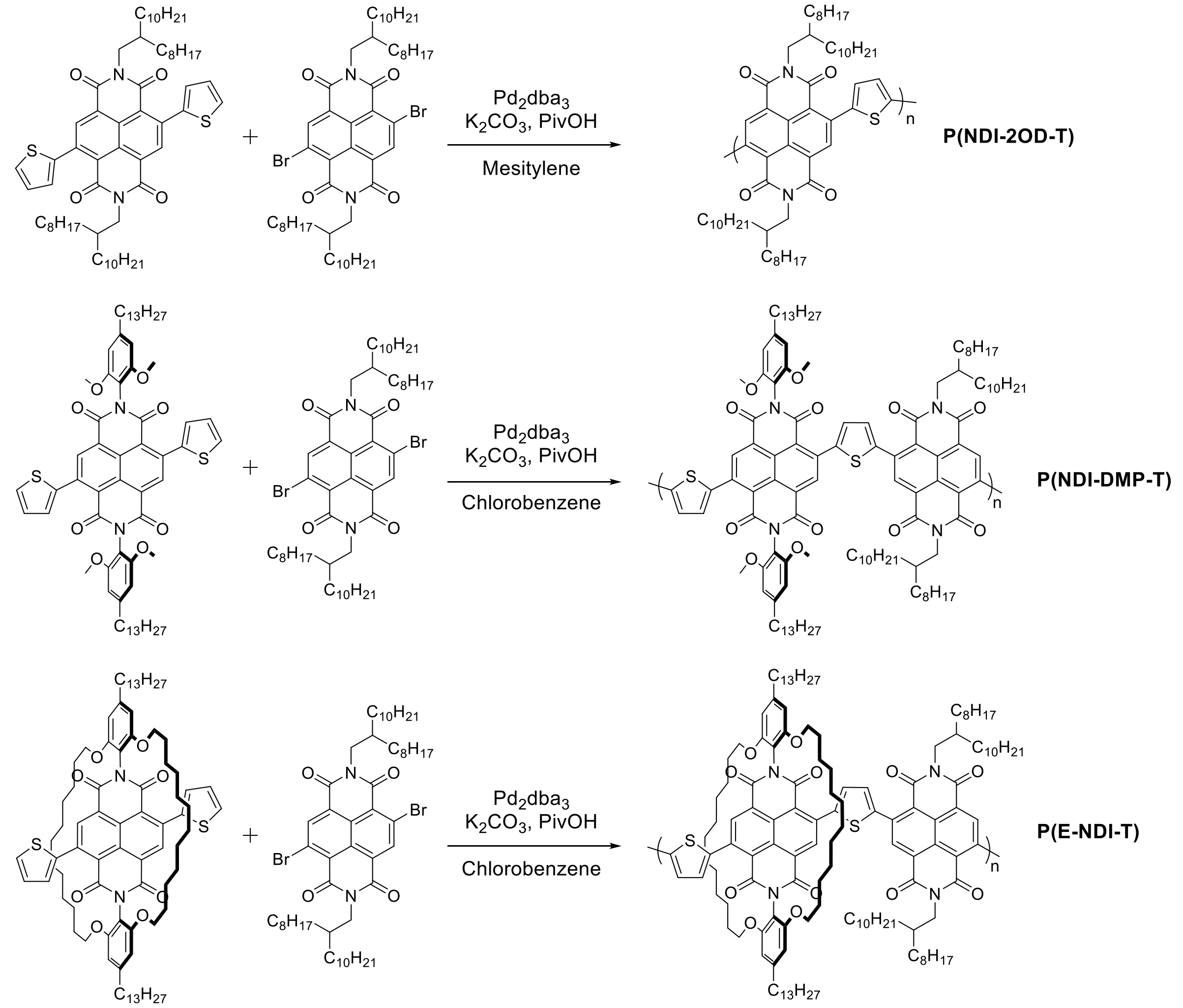

P(NDI-2OD-T)

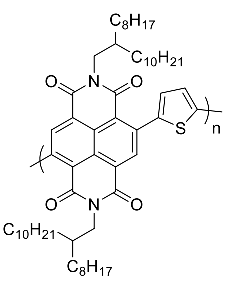

To a pre-dried μw vial under argon, Br_2-NDI (75 mg, 0.0761 mmol), encapsulated Th_2-NDI (79 mg, 0.0797 mmol, 1.05 eq.), K_2CO_3 (31.6 mg, 0.2283 mmol), PivOH (7.8 mg, 0.0761 mmol) and Pd_2dba_3 (0.7 mg, $7.64x10^{-4}$ mmol) were added and it was degassed for 30 minutes. Next, degassed mesitylene

(1.3 mL) was added and the mixture was stirred for 5 minutes at room temperature. The reactrion was placed into a pre-heated (120 ºC) oil bath and left stirring for 48 h. After cooling to room temperature, the polymer was precipitated into stirring methanol (200 mL), filtered and purified by soxhlet extraction with acetone (100 ºC) and chloroform (100 ºC). The chloroform fraction was concentrated *in vacuo,* precipitated in stirring methanol (~150 mL) and collected by filtration to afford a purple-coloured polymer (120.8 mg, 0.0665 mmol, 87%); Mn = 16.1 kg/mol, Mw = 42.2 kg/mol, Đ = 2.62.

P(NDI-DMP-T)

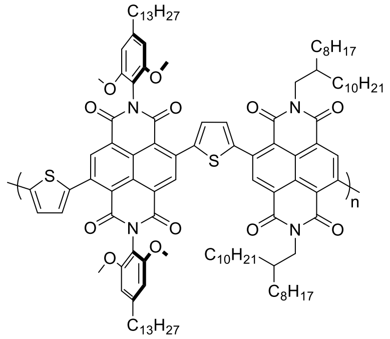

To a pre-dried µw vial under argon, Br_2-NDI (75 mg, 0.0761 mmol), Th2-NDI-OMe (81.2 mg, 0.0761 mmol), K_2CO_3 (31.6 mg, 0.2286 mmol), PivOH (7.8 mg, 0.0764 mmol) and anhydrous chlorobenzene (0.2 mL) were added. The mixture was stirred for 10 minutes at room temperature. Next, Pd_2dba_3 (0.7 mg, 0.7644 µmol) was added and the mixture was placed into a pre-heated (100 ºC) oil bath and left stirring for 20 h. After cooling to room temperature, the polymer was dissolved in the minimal amount of chlorobenzene, precipitated into stirring methanol (200 mL), filtered, and purified by soxhlet extraction with acetone (100 ºC), hexane (100 ºC) and chloroform (100 ºC). The chloroform fraction was concentrated *in vacuo*, dissolved in the minimal amount of chlorobenzene, and precipitated into stirring methanol (200 mL) to afford a purple-coloured polymer (48.1 mg, 0.0254 mmol, 33%); Mn = 16.3 kg/mol, Mw = 29.5 kg/mol, Đ = 1.81.

P(E-NDI-T)

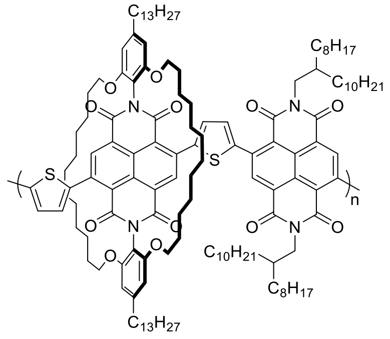

To a pre-dried µw vial under argon, Br_2-NDI (55 mg, 0.0558 mmol), encapsulated Th_2-NDI (75 mg, 0.0558 mmol), K_2CO_3 (23.2 mg, 0.1675 mmol), PivOH (5.7 mg, 0.0558 mmol) and Pd_2dba_3 (0.5 mg, 5.46×10^{-4} mmol) were added and it was degassed for 30 minutes. Next, anhydrous chlorobenzene (0.15

mL) was added, and the mixture was stirred for 5 minutes at room temperature. The mixture was then placed into a pre-heated)100 ºC) oil bath and left stirring for 14 h. After cooling to room temperature, the polymer was dissolved in the minimal amount of chlorobenzene, precipitated into stirring methanol (200 mL), filtered, and purified by soxhlet extraction with acetone (100 ºC), hexane (100 ºC) and chloroform (100 ºC). The chloroform fraction was concentrated *in vacuo*, dissolved in the minimal amount of chlorobenzene, and precipitated into stirring methanol (200 mL) to afford a red-purple coloured polymer (79.8 mg, 0.0368 mmol, 66%); Mn = 14.6 kg/mol, Mw = 38.4 kg/mol, Đ = 2.46.

NMR Spectra

Br_2-NDA

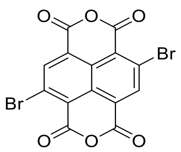

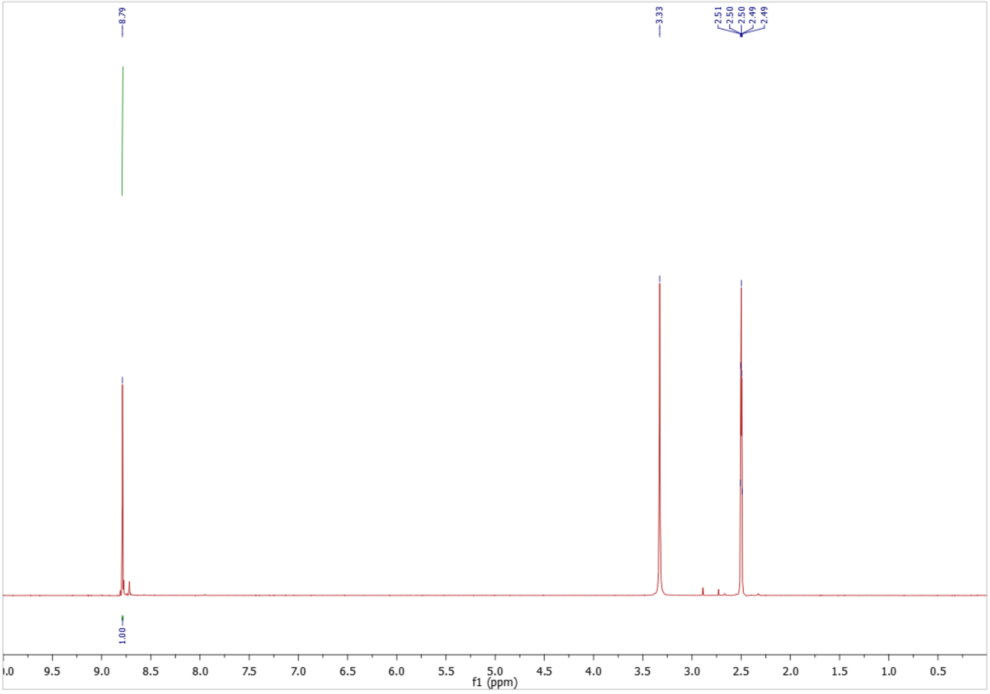

Th_2-NDA

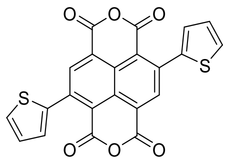

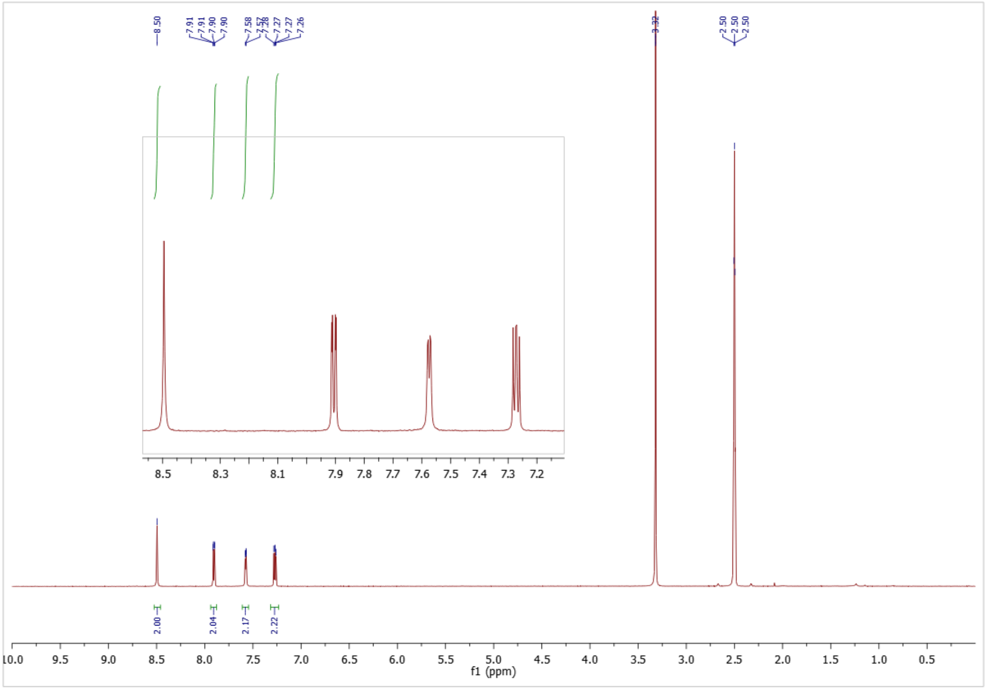
8.50
7.91
7.91
7.90
7.90
7.58
7.57
7.28
7.27
7.27
7.26
3.32
2.50
2.50
2.50
2.00
2.04
2.17
2.22
f1 (ppm)

Th_2-NDI-OMe

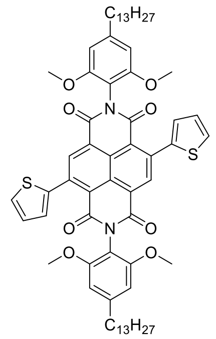

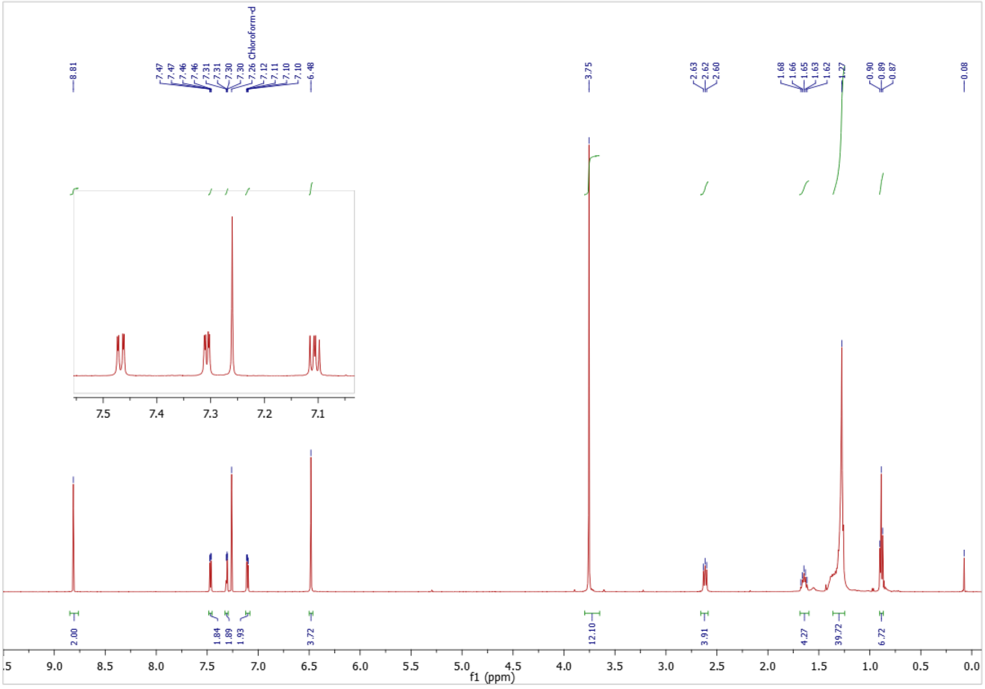

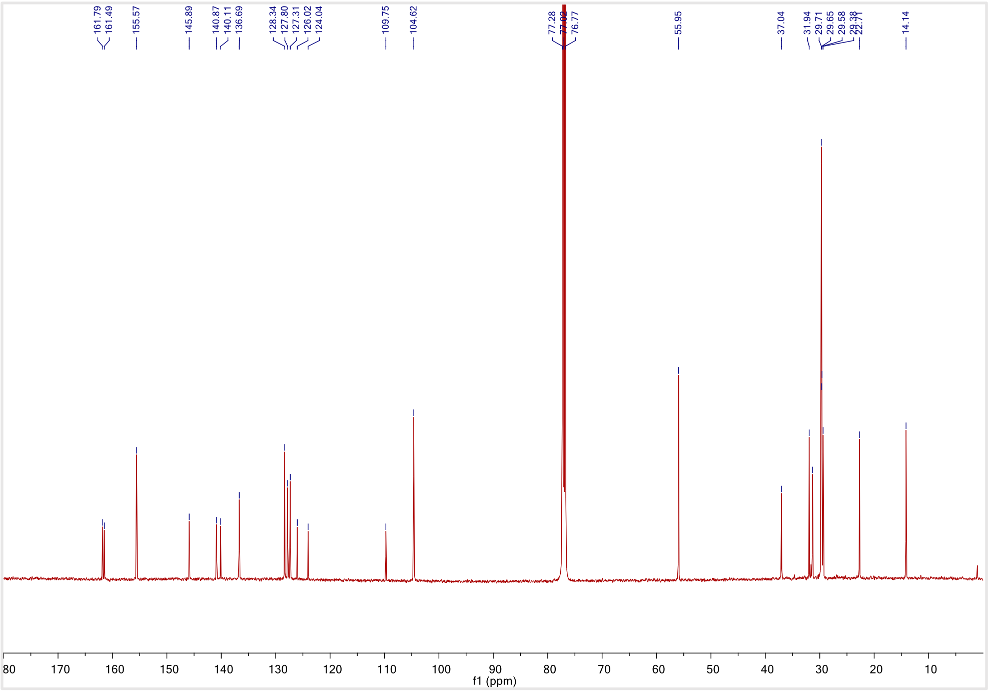

Th_2-NDI-OH

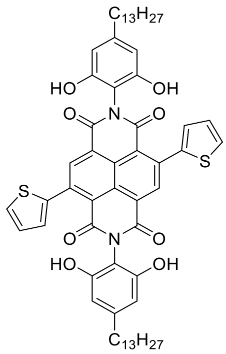
C13H27
HO
OH
O
N
O
S
S
O
N
O
HO
OH
C13H27

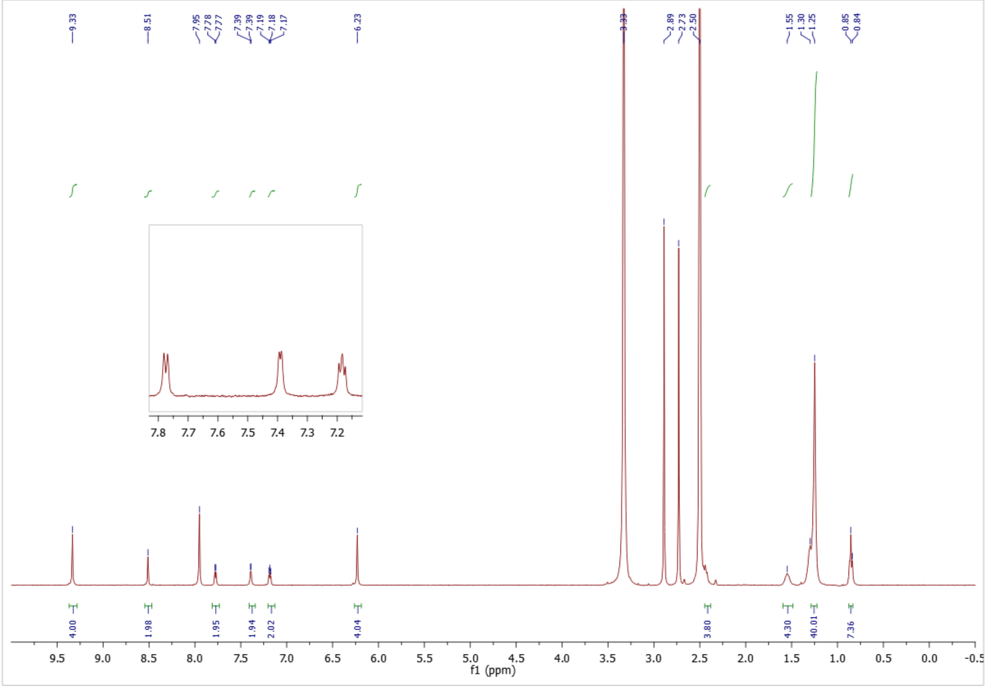
9.33
8.51
7.95
7.78
7.77
7.39
7.39
7.19
7.18
7.17
6.23
3.33
2.89
2.73
2.50
1.55
1.30
1.25
0.85
0.84
4.00
1.98
1.95
1.94
2.02
4.04
3.80
4.30
40.01
7.36
f1 (ppm)

Encapsulated NDI Monomer (E-NDI-T)

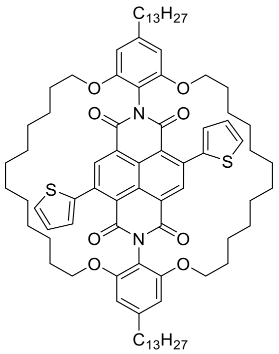

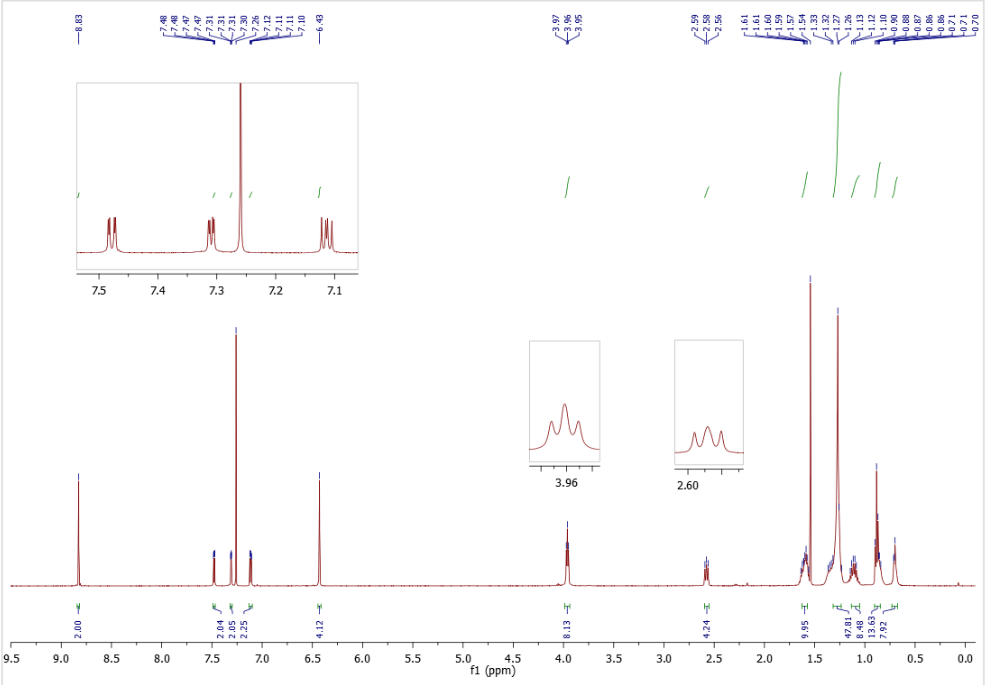

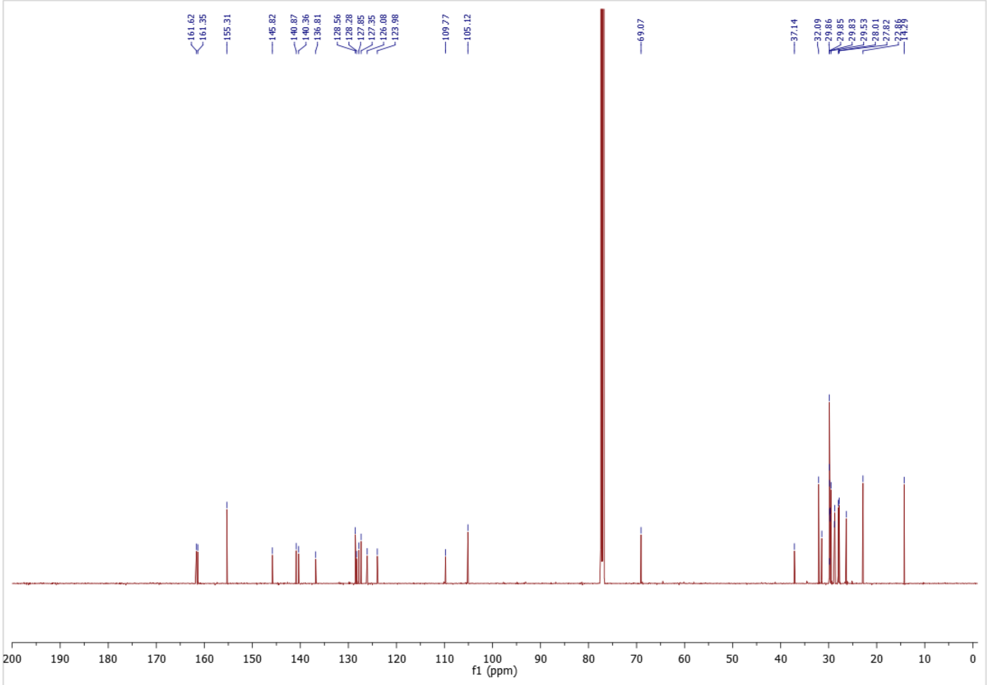

N,N′-bis(2-octyldodecyl)-2,6-dibromo-1,4,5,8-naphthalene diimide (Br_2-NDI-2OD)

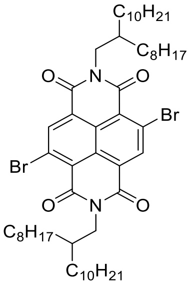

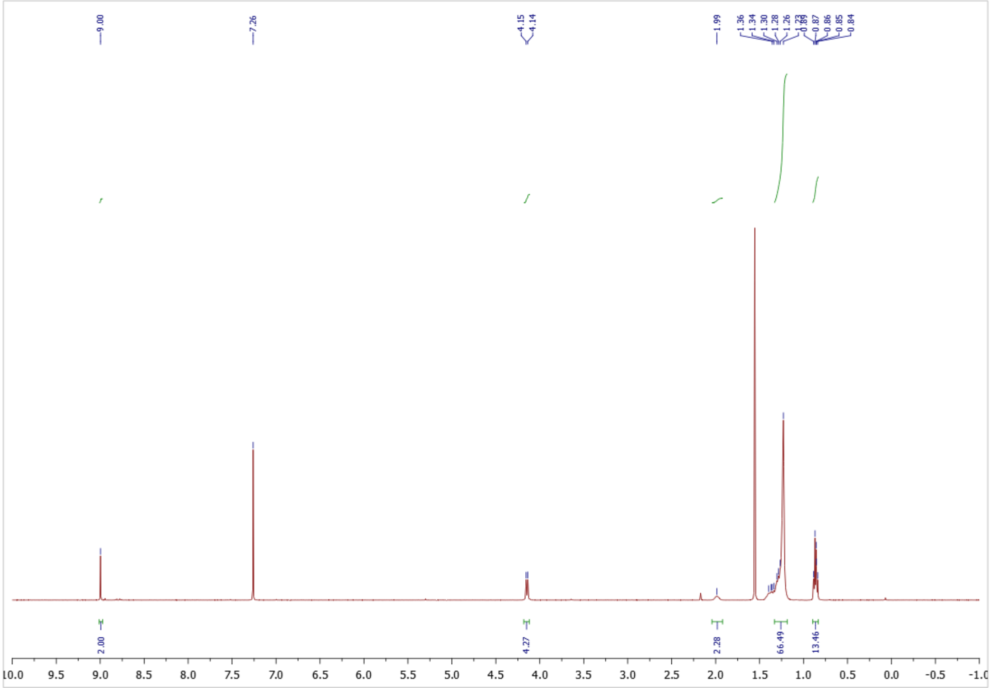

N,N'-bis(2-octyldodecyl)-2,6-bis(thiophen-2-yl)-1,4,5,8-naphthalene diimide (Th$_2$-NDI-2OD)

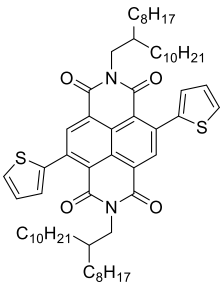

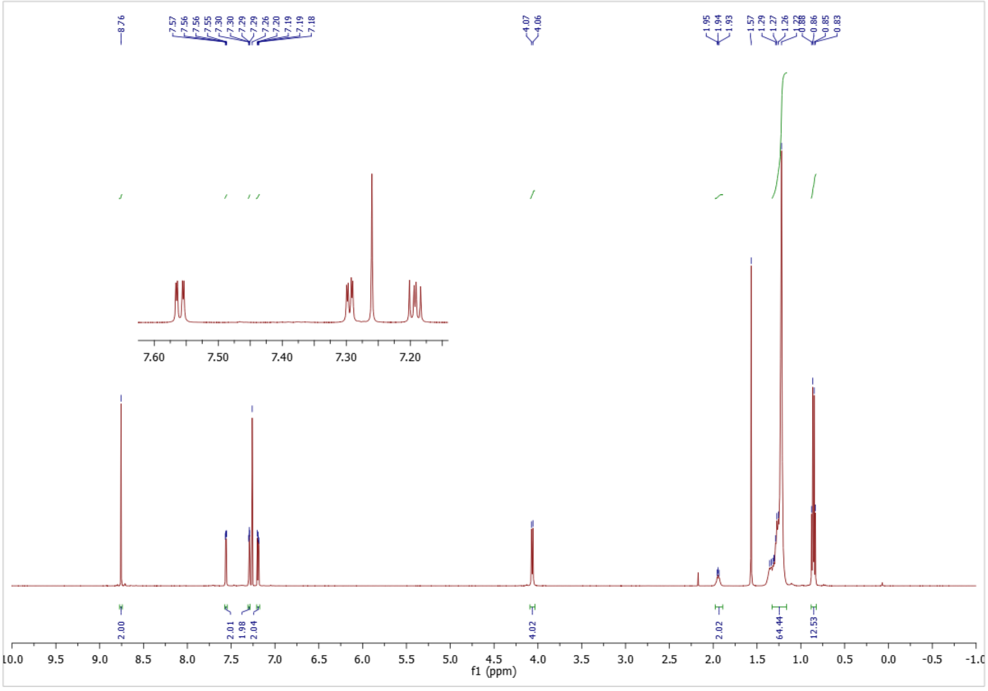

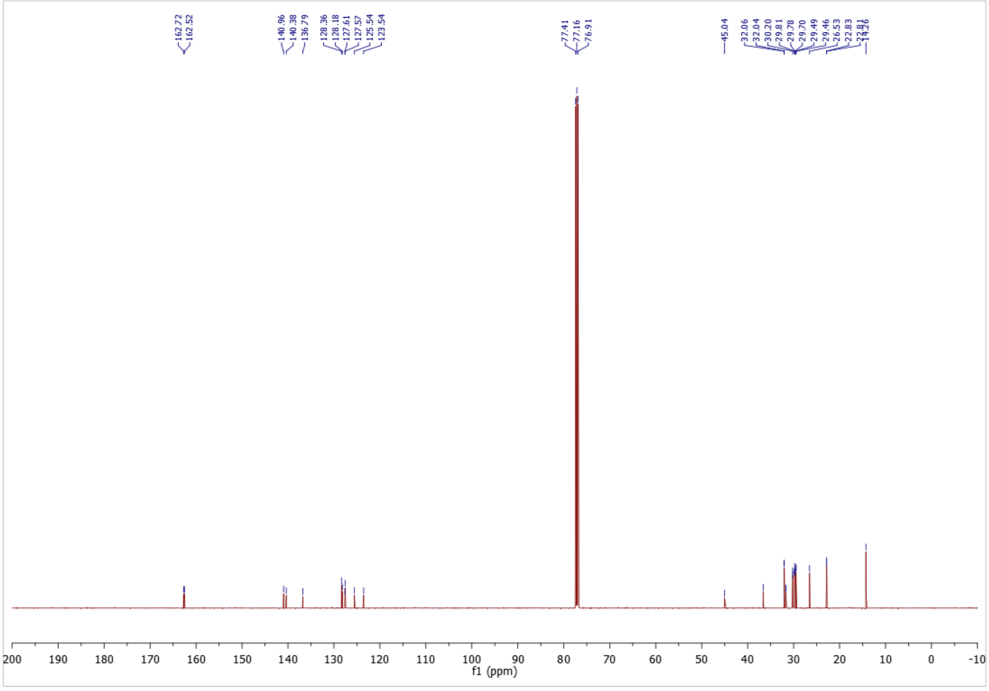

N,N'-bis(2-octyldodecyl)-2,6-bis(5-bromothiophen-2-yl)-1,4,5,8-naphthalene diimide (Br_2Th_2-NDI-2OD)

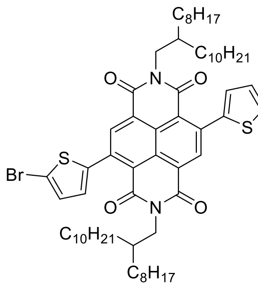

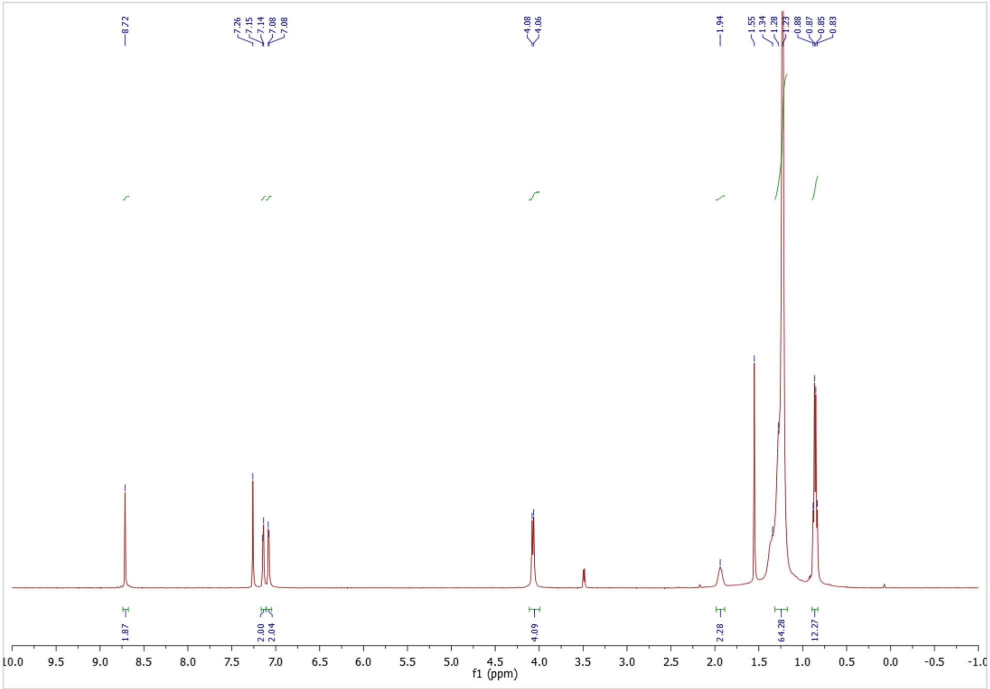

P(NDI-2OD-T)

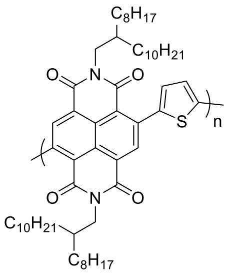
C8H17
C10H21
O
N
O
S
n
O
N
O
C10H21
C8H17

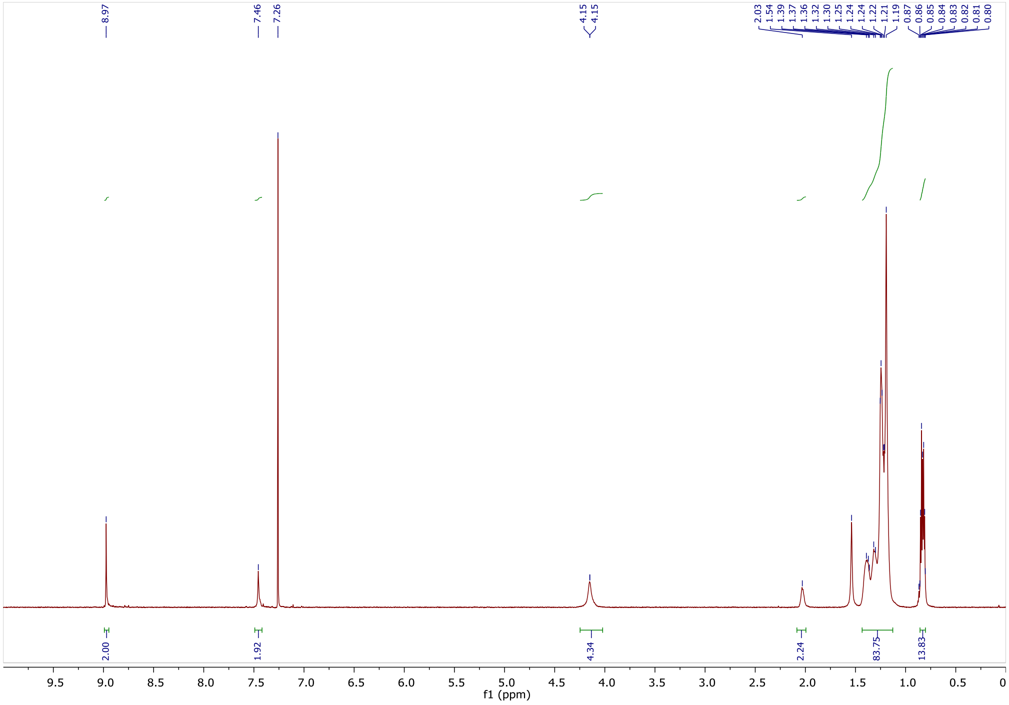
8.97
7.46
7.26
4.15
4.15
2.03
1.54
1.39
1.37
1.36
1.32
1.30
1.25
1.24
1.24
1.22
1.21
1.19
0.87
0.86
0.85
0.84
0.83
0.82
0.81
0.80
2.00
1.92
4.34
2.24
83.75
13.83
9.5
9.0
8.5
8.0
7.5
7.0
6.5
6.0
5.5
5.0
4.5
4.0
3.5
3.0
2.5
2.0
1.5
1.0
0.5
0
f1 (ppm)

P(NDI-DMP-T)

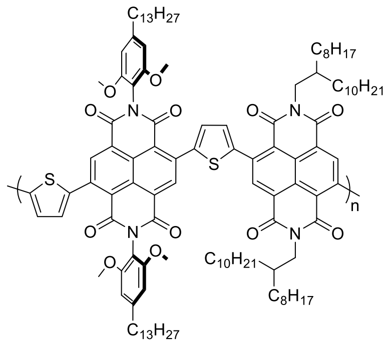

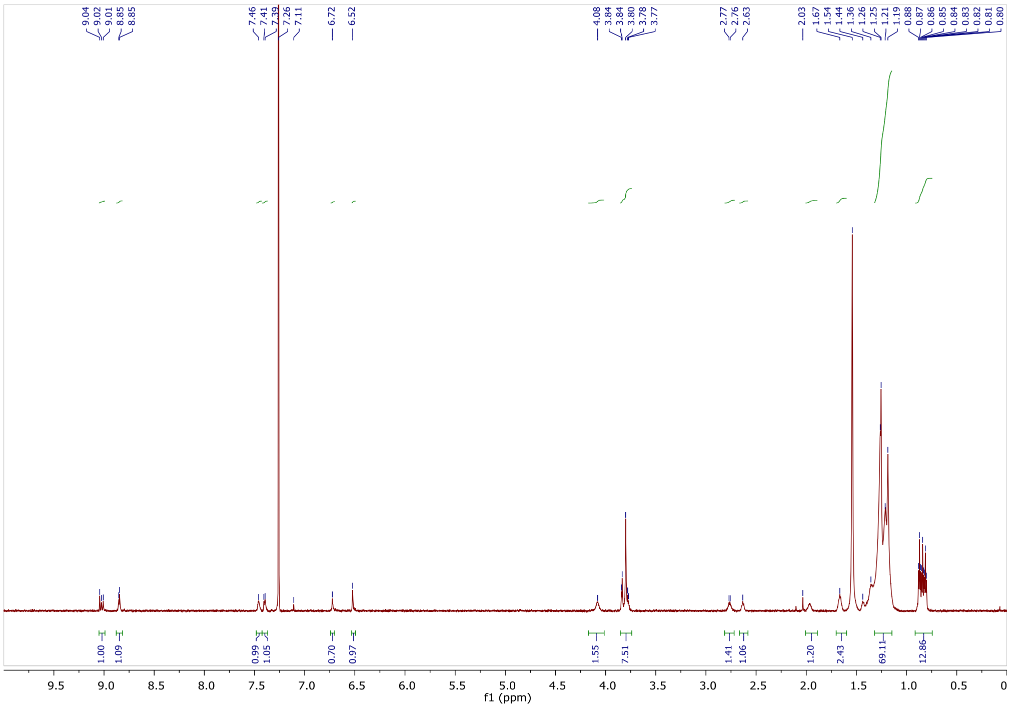

P(E-NDI-T)

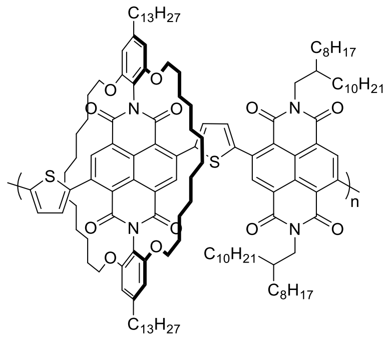

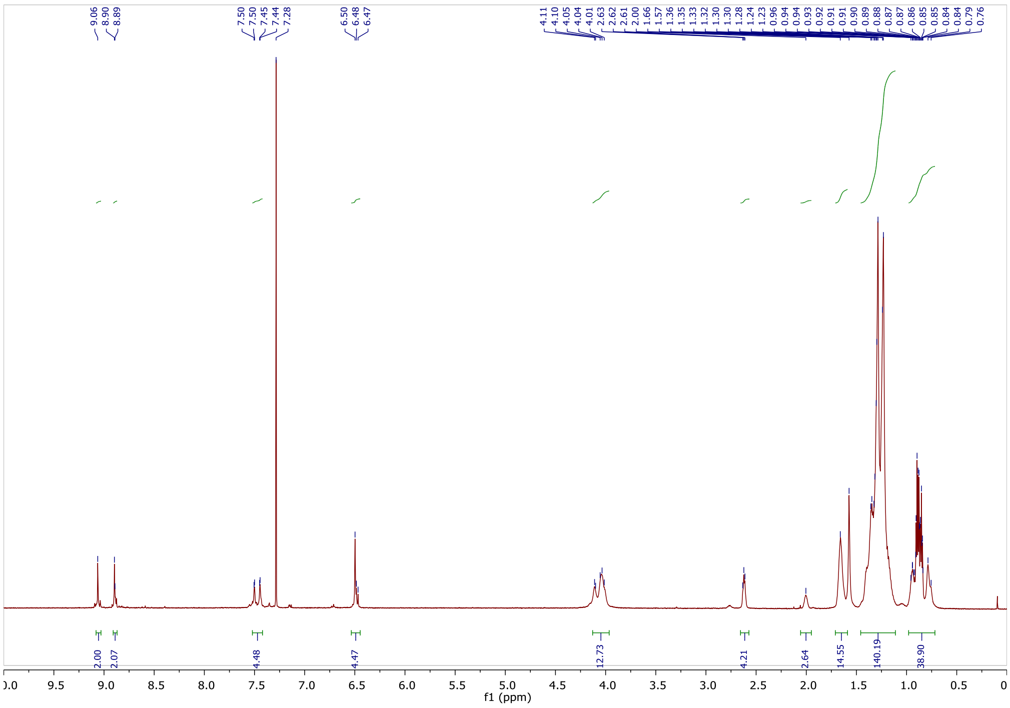

GPC Traces

P(NDI-2OD-T)

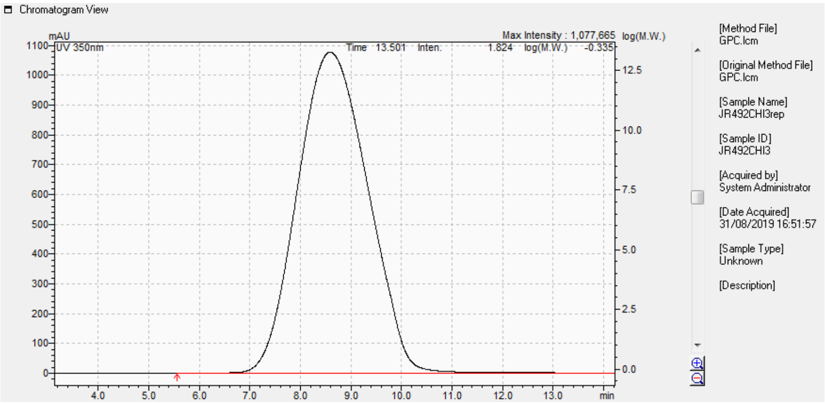

P(NDI-DMP-T)

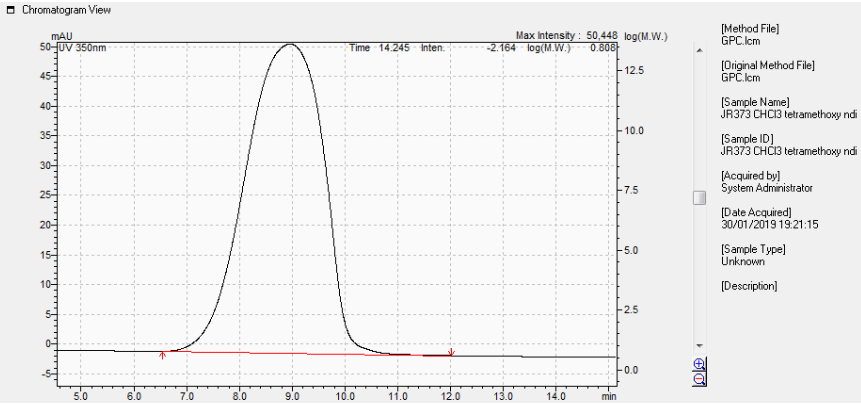

P(E-NDI-T)

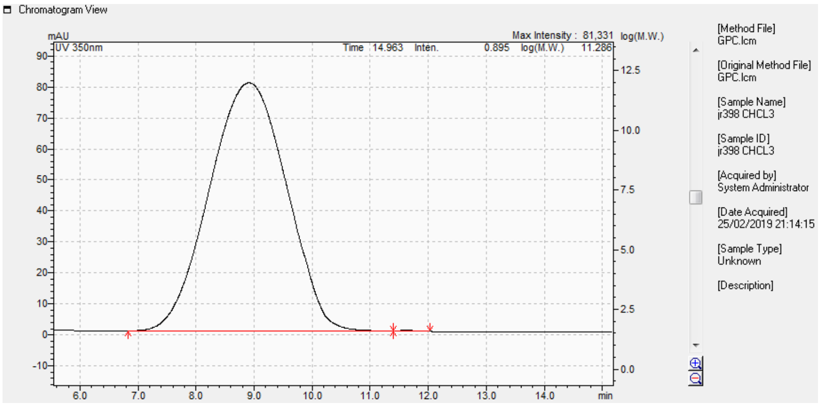

HOMO and LUMO Distributions

The following data were calculated using B3LYP/6-31G.*

P(NDI-2OD-T)

HOMO (-5.88 eV)

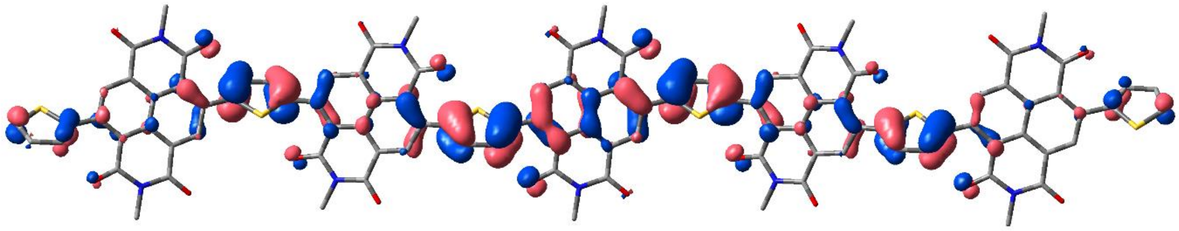

LUMO (-3.59 eV)

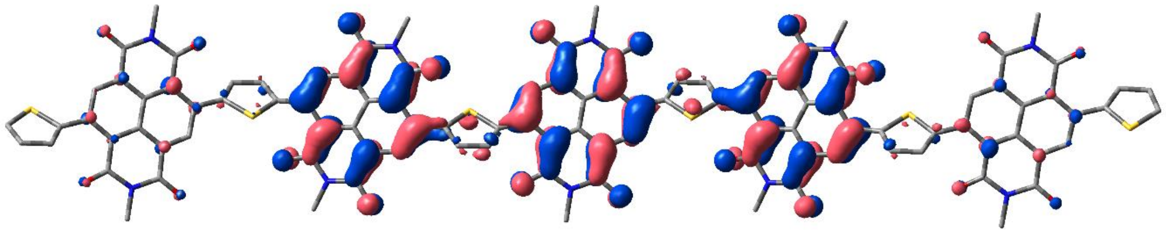

P(NDI-DMP-T)

HOMO (-5.63 eV)

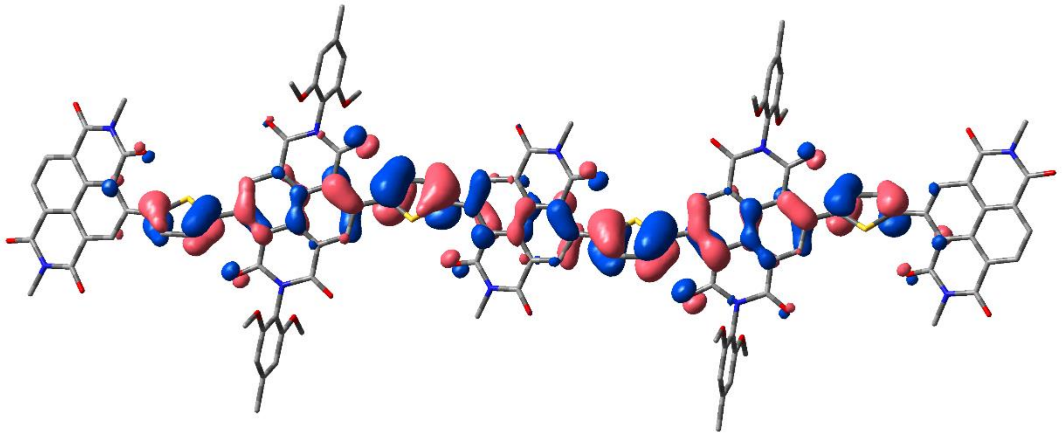

LUMO (-3.36 eV)

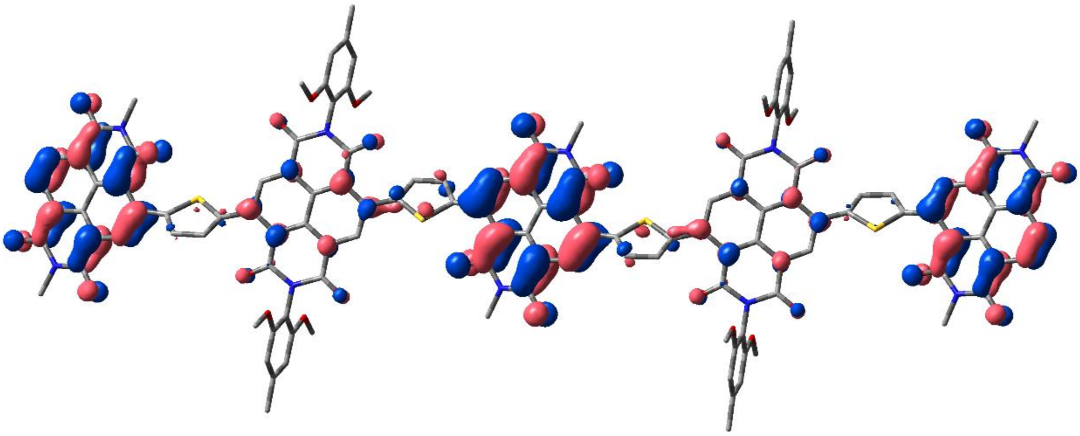

<u>P(E-NDI-T)</u>

HOMO (-5.60 eV)

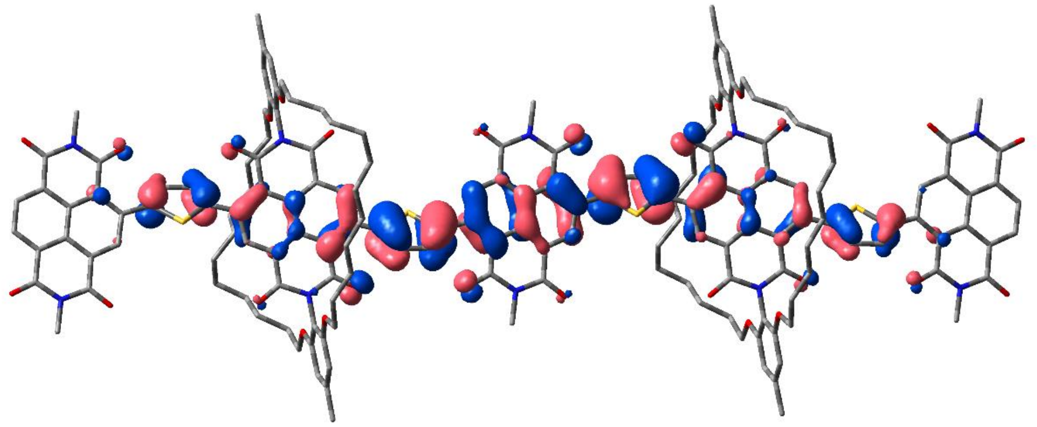

LUMO (-3.40 eV)

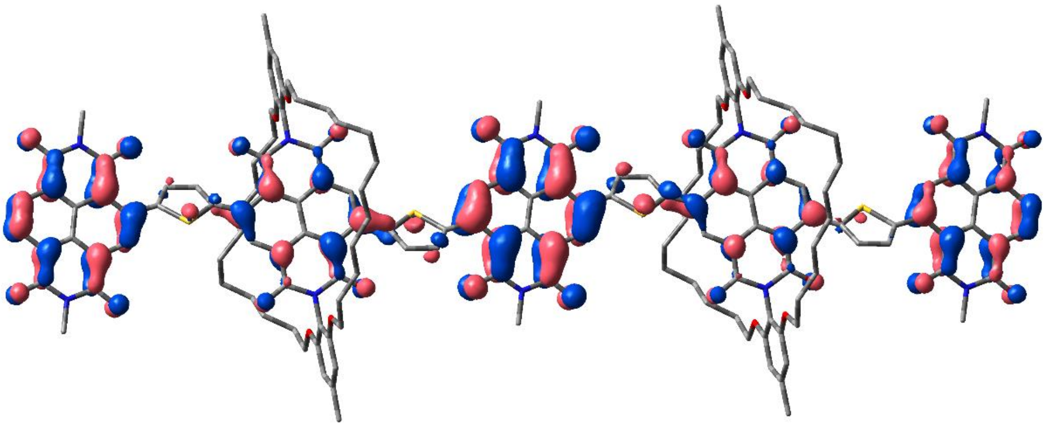

TD-DFT

Table S1. TD-DFT data of **P(NDI-2OD-T)** calculated using the B3LYP/6-31G* dataset.

No	Transition	Energy (cm^{-1})	Wavelength (nm)	Osc. strength	Orbital contributions	Dominant contribution
**1**	$S_0 \rightarrow T_1$	12295.92	813.2778	0	HOMO → LUMO (55%), H-1 → L+1 (11%)	π → π*
**2**	$S_0 \rightarrow T_2$	12713.72	786.552	0	H-1 → LUMO (24%), H-1 → L+2 (14%), HOMO → L+1 (31%)	ICT
**3**	$S_0 \rightarrow T_3$	12973.43	770.8063	0	H-2 → LUMO (19%), H-1 → L+1 (21%), HOMO → L+2 (26%)	ICT
**4**	$S_0 \rightarrow T_4$	13796.92	724.7994	0	H-3 → LUMO (14%), H-1 → L+2 (11%), H-1 → L+4 (10%), HOMO → L+3 (21%)	ICT
**5**	$S_0 \rightarrow T_5$	14240.52	702.2213	0	H-2 → L+2 (12%), H-1 → L+3 (19%), HOMO → L+4 (29%)	ICT
**6**	$S_0 \rightarrow S_1$	15130.15	660.9318	1.7817	HOMO → LUMO (76%), H-1 → L+1 (13%)	π → π*
**7**	$S_0 \rightarrow T_6$	15838.31	631.3805	0	H-5 → LUMO (22%), H-4 → L+1 (10%), H-3 → LUMO (12%), H-3 → L+2 (10%), H-2 → L+1 (12%), H-2 → L+3 (10%), H-1 → L+4 (10%)	ICT
**8**	$S_0 \rightarrow S_2$	15990.75	625.3616	0.0083	H-1 → LUMO (37%), HOMO → L+1 (49%)	ICT

Table S2. TD-DFT data of **P(NDI-DMP-T)** calculated using the B3LYP/6-31G* dataset.

No	Transition	Energy (cm^{-1})	Wavelength (nm)	Osc. strength	Orbital contributions	Dominant contribution
**1**	$S_0 \rightarrow T_1$	12283.82	814.0787	0	HOMO → LUMO (51%), HOMO → L+2 (11%)	π → π*
**2**	$S_0 \rightarrow T_2$	12810.5	780.6094	0	H-1 → LUMO (31%), HOMO → L+1 (20%), HOMO → L+3 (15%)	ICT
**3**	$S_0 \rightarrow T_3$	13336.38	749.8288	0	H-6 → LUMO (15%), H-1 → L+1 (23%), HOMO → L+2 (30%)	ICT
**4**	$S_0 \rightarrow T_4$	14139.71	707.2283	0	H-11 → LUMO (10%), H-6 → L+1 (11%), H-1 → L+2 (24%), HOMO → L+3 (17%)	ICT
**5**	$S_0 \rightarrow T_5$	14629.28	683.5604	0	H-1 → L+3 (22%), HOMO → L+4 (30%)	ICT
**6**	$S_0 \rightarrow S_1$	15091.44	662.6273	1.404	HOMO → LUMO (74%), HOMO → L+2 (11%)	π → π*
**7**	$S_0 \rightarrow T_6$	15846.37	631.0592	0	H-11 → L+2 (11%), H-1 → LUMO (19%), H-1 → L+2 (13%), H-1 → L+4 (16%)	ICT
**8**	$S_0 \rightarrow S_2$	16105.28	620.9144	0.0051	H-1 → LUMO (38%), HOMO → L+1 (47%)	ICT

Table S3. TD-DFT data of **P(E-NDI-T)** calculated using the B3LYP/6-31G* dataset.

No	Transition	Energy (cm^{-1})	Wavelength (nm)	Osc. strength	Orbital contributions	Dominant contribution
**1**	$S_0 \rightarrow T_1$	11593.41	862.5587	0	HOMO → LUMO (58%), HOMO → L+2 (15%)	π → π*
**2**	$S_0 \rightarrow T_2$	12454.01	802.9544	0	H-1 → LUMO (32%), HOMO → L+1 (24%)	ICT
**3**	$S_0 \rightarrow T_3$	12885.51	776.0653	0	H-6 → LUMO (13%), H-1 → L+1 (28%), HOMO → L+2 (23%)	ICT
**4**	$S_0 \rightarrow T_4$	13950.17	716.8374	0	H-11 → LUMO (15%), H-1 → L+2 (22%), HOMO → L+3 (22%)	ICT
**5**	$S_0 \rightarrow T_5$	14577.66	685.9809	0	H-1 → L+3 (23%), HOMO → L+4 (35%)	ICT
**6**	$S_0 \rightarrow S_1$	14756.72	677.6574	1.8818	HOMO → LUMO (81%)	π → π*
**7**	$S_0 \rightarrow T_6$	15896.38	629.074	0	H-11 → LUMO (13%), H-11 → L+2 (14%), H-1 → LUMO (13%), H-1 → L+4 (17%)	ICT
**8**	$S_0 \rightarrow S_2$	16107.7	620.8212	0.0032	H-1 → LUMO (27%), HOMO → L+1 (60%)	ICT

AIEE Study

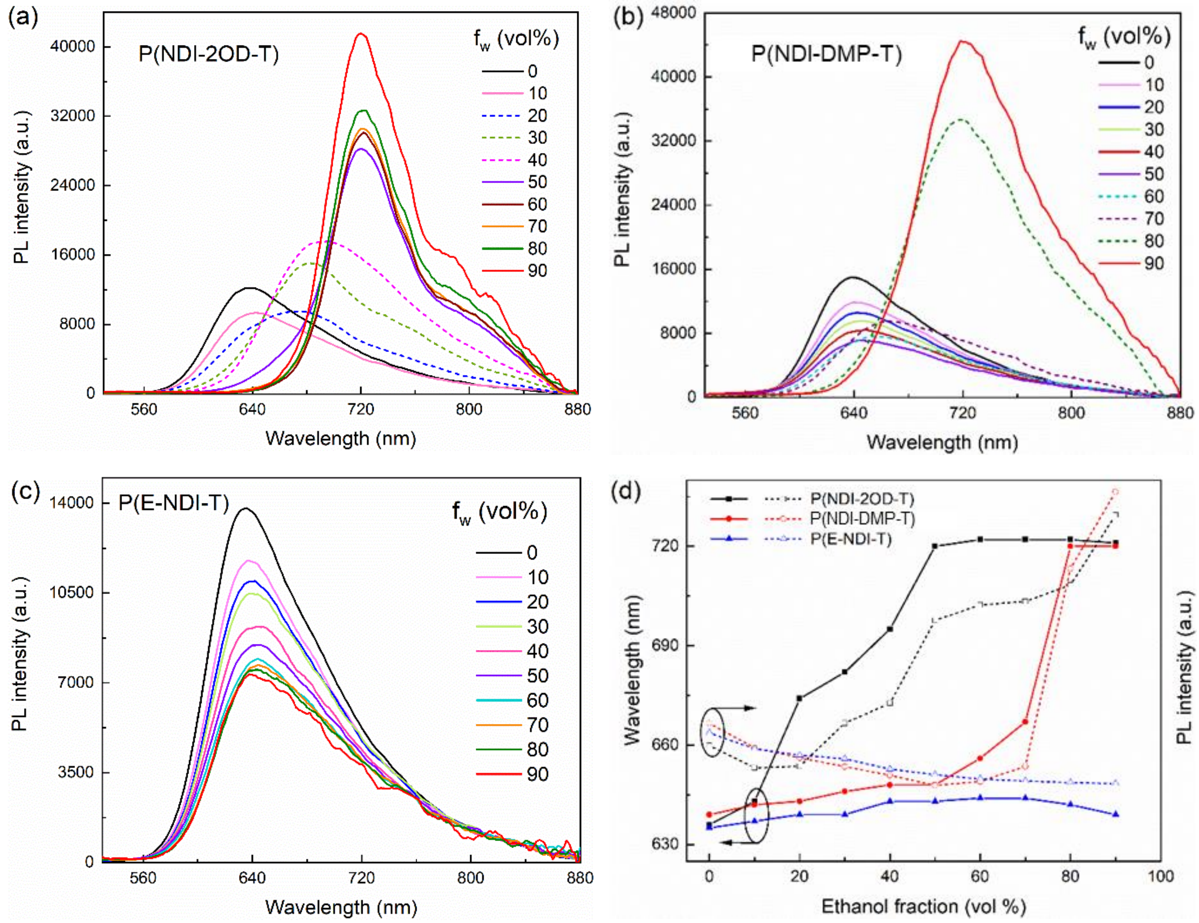

Figure S1. Emission spectra of **P(NDI-2OD-T)** (a), **P(NDI-DMP-T)** (b) and **P(E-NDI-T)** (c) in $CHCl_3$/ethanol mixtures with different ethanol fractions (f_w). The excitation wavelength was 375 nm in all cases. (d) Plots of maximum emission intensity and wavelength of the NDI-T copolymers versus f_w.

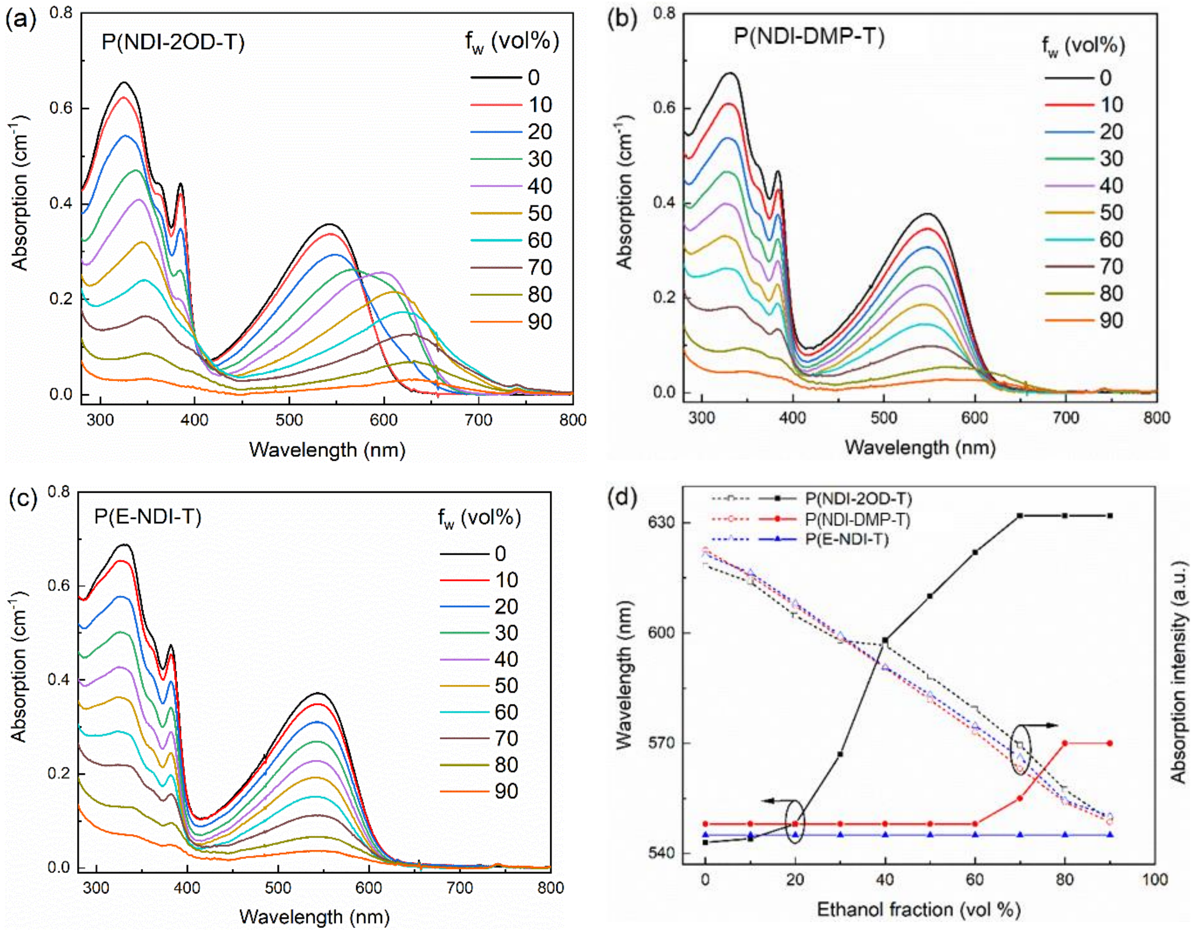

Figure S2. Absorption spectra of **P(NDI-2OD-T)** (a), **P(NDI-DMP-T)** (b) and **P(E-NDI-T)** (c) in $CHCl_3$/ethanol mixtures with different ethanol fractions (the initial $CHCl_3$ concentration was 0.01 g L^{-1}). (d) Plots of the maxima absorption intensity and wavelength in the long wavelength region of the NDI-T copolymers versus f_w.

Transient Photoluminescence Lifetimes

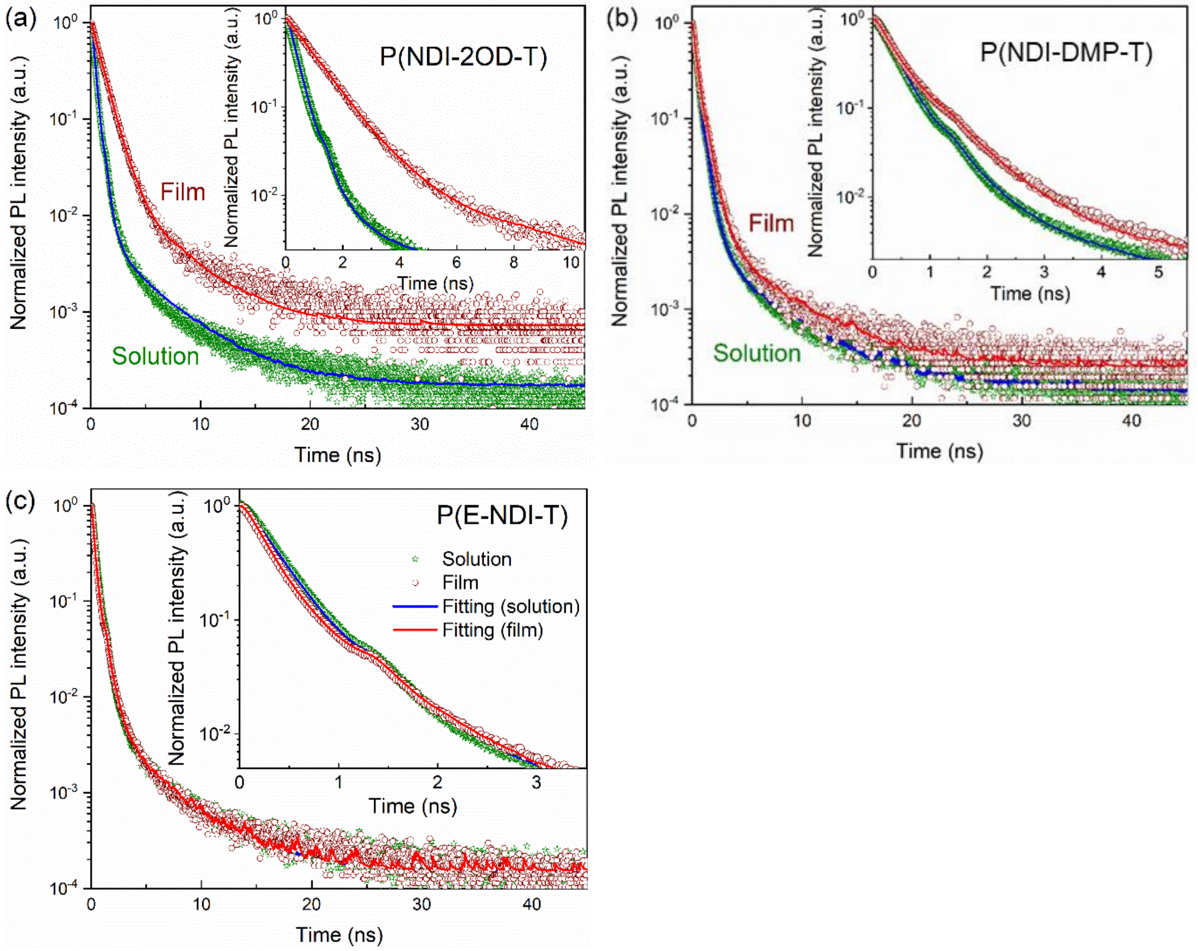

Figure S3. Semi-log plot of the transient PL (measured at the wavelength at which the fluorescence emission is peaked) for (a) **P(NDI-2OD-T)**, (b) **P(NDI-DMP-T)** and (c) **P(E-NDI-T)** in $CHCl_3$ solution and thin film. All transient spectra were excited at 375 nm with a ps pulsed laser in air at room temperature. The solid line are the fits to the experimental data a (hollow symbols) obtained via iterative re-convolution of an n-exponential function with the instrument response.

X-Ray Crystallography

X-ray crystallographic data were collected for **E-NDI-T** using a D8-QUEST PHOTON-100 diffractometer equipped with an Incoatec IμS Cu microsource. The temperature was held at 180(2) K using an Oxford Cryosystems N_2 cryostat. Data integration and reduction were carried out with *SAINT* in the *APEX3* software suite. A multi-scan correction was applied using *SADABS*. Structures were solved using *SHELXT* and refined using *SHELXL*.[7,8] The molecules display inversion symmetry and are situated on inversion centres in space group $P2_1/c$. The structure exhibits some disorder, which was treated as follows:

- The thiazole ring was modelled as two components, related by a 180 degree rotation around the C7–C8 bond. The refined site occupancies of the two components are 0.619(3):0.381(3). Both S atoms were refined with anisotropic ADPs. The C atoms in both disorder components are refined with a common isotropic displacement parameter (one parameter applied to six C atom sites). The geometry of the minor component was tied to that of the major component using a SAME restraint.
- The middle atoms of the $–(CH_2)_{12}–$ alkyl strap (C23 and C24) were modelled as two components with refined site occupancies 0.633(6):0.367(6). The geometry was restrained with standard C–C and C…C distances, and ISOR restraints were applied to control the anisotropic ADPs of the split C atoms.

CCDC number	2079163
Cambridge data number	HB_B1_0001
Chemical formula	$C_{84}H_{114}N_2O_8S_2$
Formula weight	1343.89
Temperature / K	180(2)
Crystal system	monoclinic
Space group	$P2_1/c$
a / Å	15.2329(5)
b / Å	21.4306(7)
c / Å	12.4106(4)
α / °	90
β / °	109.796(2)
γ / °	90
Unit-cell volume / $Å^3$	3812.0(2)
Z	2
Calc. density / g cm^{-3}	1.171
F(000)	1456
Radiation type	CuKα (λ_{ave} = 1.5418 Å)
Absorption coefficient / mm^{-1}	1.067
Crystal size / mm^3	0.15 x 0.10 x 0.02
2θ range / °	6.17-133.49
Completeness to max 2θ	0.997
No. of reflections measured	35932
No. of independent reflections	6726
R(int)	0.1382
No. parameters / restraints	454 / 48
Final R1 values ($I > 2\sigma(I)$)	0.0669
Final wR(F^2) values (all data)	0.1315
Goodness-of-fit on F^2	1.020
Largest difference peak & hole / e $Å^{-3}$	0.410, -0.357

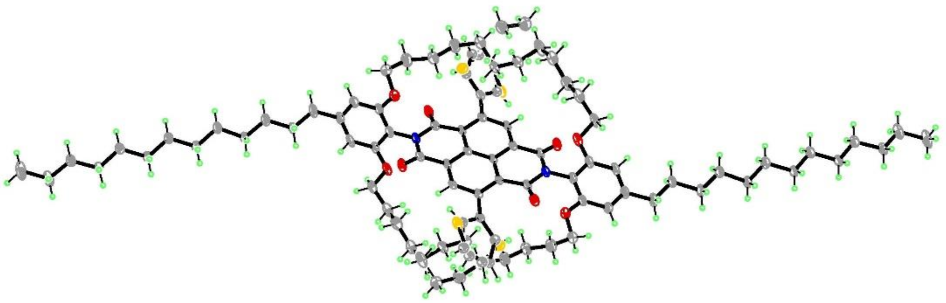

Figure S4. X-Ray crystal structure of **E-NDI-T.**

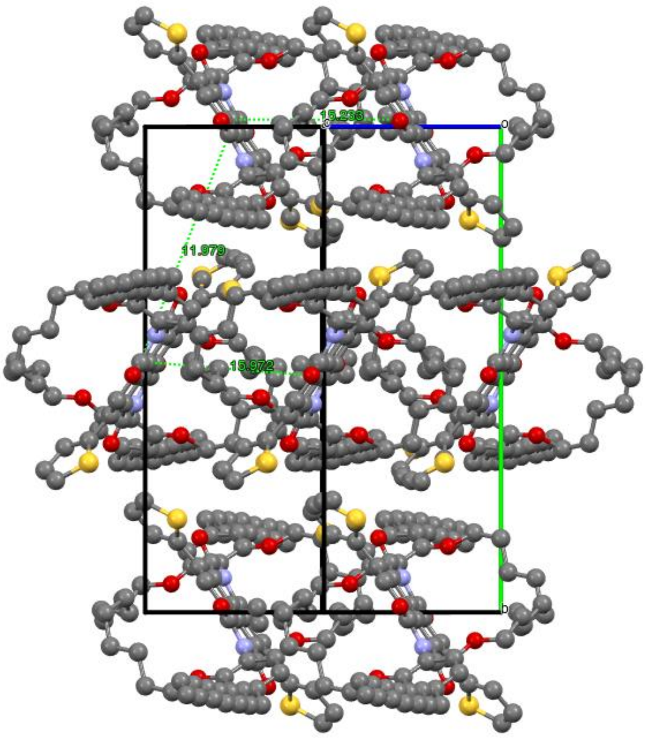

Figure S5. Three-dimensional packing of **E-NDI-T**.

Grazing Incidence X-ray Scattering studies

Grazing incidence wide X-ray scattering (GIWAXS) was performed at I07 (Diamond Light Source, Rutherford, U.K.) at an X-ray beam energy of 12.4 keV. Scattering patterns were recorded on a vertically-offset Pilatus 2M detector with a sample to detector distance of 635.62 mm, calibrated using a silver behenate standard to achieve a Q range of 0.045 – 1.8 $Å^{-1}$. Alignment was performed via three iterative height (z) and rocking curve (**Ω**) scans, with the final grazing incidence angle set to **Ω** = 0.3º. The two-dimensional scattering patterns were masked to remove the sample horizon, detector module gaps and beam-stop and radially integrated from the apparent beam centre. Data correction and reduction was performed using the GIXSGUI MATLAB toolbox.[9] Two-dimensional scattering data was reduced to one-dimensional via radial integration, which was performed with a mask to remove contributions from "hot pixels", the substrate horizon and reflected beam.

OPV Device Data

The inverted device architecture is ITO /ZnO /active laryer /MoO_3 /Ag. Pre-patterned ITO-coated glass wafers (Thin Film Devices, Inc.) with a sheet resistance of ≈20 Ω/sq were used as substrates. The ZnO precursor solution is prepared by dissolving 220 mg of zinc acetate dehydrate (Sigma Aldrich) and 62 mg of 2-ethanolamine (Sigma Aldrich) in 2 mL 2-methoxyethanol (Sigma Aldrich), then stir overnight. The ZnO precursor is spin-coated on precleaned ITO glass at 7000 rpm after filtering through a 0.45 µm PVDF filter and 20 min at 170°C in air. Then the device is transferred into an argon glove box. After that, active layer solutions (10 mg/mL in total) were then spin-coated onto the ZnO layer (Argon filled glovebox) while spinning 1500 RPM. All the substrates are loaded into a metal-evaporation chamber and mask with dimensions of 6.25 mm^2. Then, these substrates are vapor deposited molybdenum oxide interlayer (10 nm) and silver (100 nm) electrode at high vacuum (≈6 x 10^{-6} Torr). All the devices were measured under a simulated AM1.5G irradiation (100 mW cm^{-2}) illumination with a standard ABET Sun 2000 Solar Simulator in the nitrogen filled glovebox. A standard silicon solar cell was used to calibrate the light intensity. The voltage was scanned from -0.20 V to 1.20 V.

Table S4. Photovoltaic parameters (AM1.5G) for optimized solar cells employing the indicated blends.

Donor	Acceptor	V_{oc} (V)	J_{sc} (mA/cm^{-2})	FF (%)	PCE[a] (%)
**J52**	**P(NDI-2OD-T)**	0.77 ($0.77 \pm 4.1 \times 10^{-3}$)	7.52 (6.68±0.37)	54.50 (50.84±2.38)	3.14 (2.62±0.26)
	**P(NDI-DMP-T)**	0.87 ($0.86 \pm 2.2 \times 10^{-2}$)	3.97 (3.81±0.27)	45.20 (43.56±3.90)	1.57 (1.42±0.11)
	**P(E-NDI-T)**	0.87 ($0.86 \pm 2.0 \times 10^{-2}$)	3.95 (3.89±0.23)	38.59 (35.66±6.37)	1.33 (1.18±0.19)
	**P(NDI-2OD-T)**	0.77 ($0.76 \pm 5.8 \times 10^{-3}$)	8.45 (8.22±0.36)	45.42 (44.21±1.03)	2.95 (2.77±0.14)

**PTB7-Th**	**P(NDI-DMP-T)**	0.78 (0.77±4.1x10^{-2})	0.91 (0.76±0.15)	32.34 (31.41±1.73)	0.232 (0.18±3.4 x10^{-2})
	**P(E-NDI-T)**	0.78 (0.80±2.0x10^{-2})	3.78 (3.57±0.23)	37.84 (36.92±1.31)	1.10 (1.05±6.9 x10^{-2})

[a] Average of 6 champion devices. Device area = 6.25 mm^2. Active layer was annealed at 110 °C, 3 min.

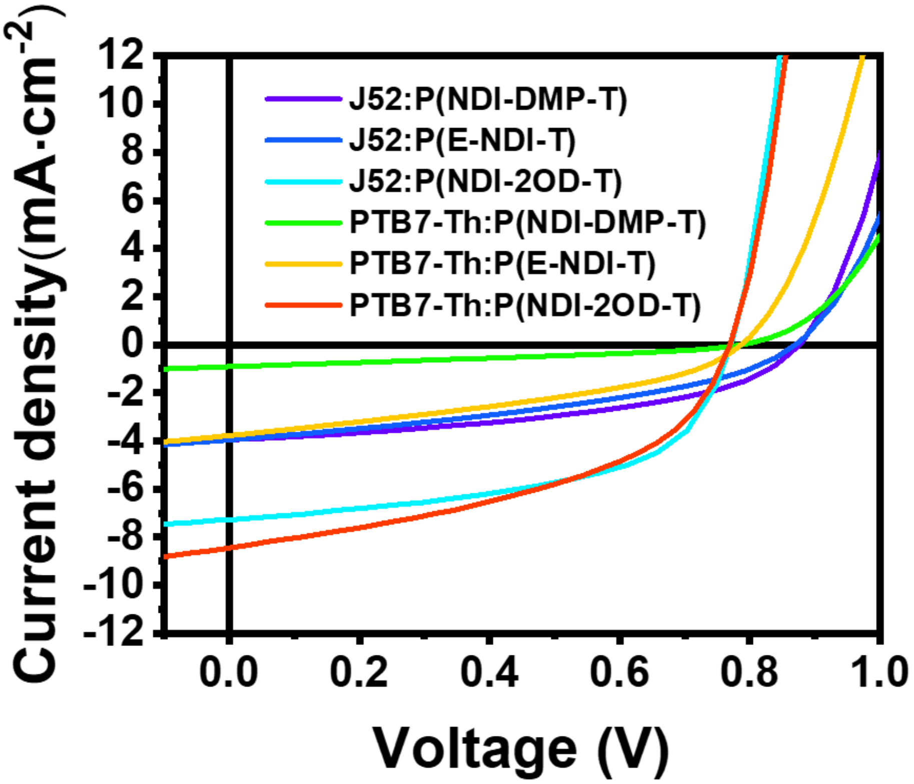

Figure S6. (a) Representative *J*–*V* curves of inverted OSCs, under AM 1.5G (100 mW cm^{-2}) illumination.

References

(1) de Mello, J. C.; Wittmann, H. F.; Friend, R. H. An Improved Experimental Determination of External Photoluminescence Quantum Efficiency. *Adv. Mater.* **1997**, *9* (3), 230–232.

(2) Fernando, R.; Etheridge, F.; Muller, E.; Sauve, G. Tuning the Optical and Electrochemical Properties of Core-Substituted Naphthalenediimides with Styryl Imide Substituent. *New J. Chem.* **2015**, *39* (4), 2506–2514. https://doi.org/10.1039/C4NJ01645A.

(3) Thalacker, C.; Röger, C.; Würthner, F. Synthesis and Optical and Redox Properties of Core-Substituted Naphthalene Diimide Dyes. *J. Org. Chem.* **2006**, *71* (21), 8098–8105. https://doi.org/10.1021/jo0612269.

(4) Bell, T. D. M.; Yap, S.; Jani, C. H.; Bhosale, S. V; Hofkens, J.; De Schryver, F. C.; Langford, S. J.; Ghiggino, K. P. Synthesis and Photophysics of Core-Substituted Naphthalene Diimides: Fluorophores for Single Molecule Applications. *Chem. – An Asian J.* **2009**, *4* (10), 1542–1550. https://doi.org/10.1002/asia.200900215.

(5) Piyakulawat, P.; Keawprajak, A.; Chindaduang, A.; Hanusch, M.; Asawapirom, U. Synthesis and Preliminary Characterization of Novel Naphthalene Bisimide Based Copolymers. *Synth. Met.* **2009**, *159* (5–6), 467–472.

(6) Senkovskyy, V.; Tkachov, R.; Komber, H.; Sommer, M.; Heuken, M.; Voit, B.; Huck, W. T. S.; Kataev, V.; Petr, A.; Kiriy, A. Chain-Growth Polymerization of Unusual Anion-Radical Monomers Based on Naphthalene Diimide: A New Route to Well-Defined n-Type Conjugated Copolymers. *J. Am. Chem. Soc.* **2011**, *133* (49), 19966–19970. https://doi.org/10.1021/ja208710x.

(7) Sheldrick, G. M. SHELXT–Integrated Space-Group and Crystal-Structure Determination. *Acta Crystallogr. Sect. A Found. Adv.* **2015**, *71* (1), 3–8.

(8) Sheldrick, G. M. Crystal Structure Refinement with SHELXL. *Acta Crystallogr. Sect. C Struct. Chem.* **2015**, *71* (1), 3–8.

(9) Jiang, Z., GIXSGUI: a MATLAB toolbox for grazing-incidence X-ray scattering data visualization and reduction, and indexing of buried three-dimensional periodic nanostructured films. *Journal of Applied Crystallography* **2015,** *48* (3), 917-926.
